# From Perception to Navigation in Environments with Persons: An Indoor Evaluation of the State of the Art

**DOI:** 10.3390/s22031191

**Published:** 2022-02-04

**Authors:** Carlos Medina Sánchez, Matteo Zella, Jesús Capitán, Pedro J. Marrón

**Affiliations:** 1 Networked Embedded Systems Group, University of Duisburg-Essen, 45127 Essen, Germany; matteo.zella@uni-due.de (M.Z.); pjmarron@uni-due.de (P.J.M.); 2 Department of Systems Engineering and Automation, Higher Technical School of Engineering, University of Seville, 41092 Seville, Spain; jcapitan@us.es

**Keywords:** motion prediction, social navigation, service robotics, path planning, sensors

## Abstract

Research in the field of social robotics is allowing service robots to operate in environments with people. In the aim of realizing the vision of humans and robots coexisting in the same environment, several solutions have been proposed to (1) perceive persons and objects in the immediate environment; (2) predict the movements of humans; as well as (3) plan the navigation in agreement with socially accepted rules. In this work, we discuss the different aspects related to social navigation in the context of our experience in an indoor environment. We describe state-of-the-art approaches and experiment with existing methods to analyze their performance in practice. From this study, we gather first-hand insights into the limitations of current solutions and identify possible research directions to address the open challenges. In particular, this paper focuses on topics related to perception at the hardware and application levels, including 2D and 3D sensors, geometric and mainly semantic mapping, the prediction of people trajectories (physics-, pattern- and planning-based), and social navigation (reactive and predictive) in indoor environments.

## 1. Introduction

Unlike a few years ago, the presence of robots in social environments has become commonplace. Nowadays, it is possible to find robots that assist elderly people [1], others that help in teaching in educational institutions [2], robots that guide people at airports [3] and museums [4], robots that perform mail delivery and basic tasks in offices [5], and robots that perform cleaning tasks in homes such as the Roomba [6]. For this reason, the number of research papers trying to address issues related to social robotics has been increasing considerably. Of course, the number of subtopics within the field of social robotics can be very broad and some papers address more specific topics than others. In this paper, we aimed to present a qualitative and, in some cases, a quantitative analysis of the elements involved in social navigation in indoor environments: from perception, through mapping, the prediction of people trajectories and finally, the specific topic of social navigation, which we consider to be part of the flow of elements that need to be addressed in order to develop socially acceptable robots coexisting with people. We discuss each topic by presenting the most prominent and recent related works.

*Perception* is the starting point to implement correct navigation. Nowadays, there is a wide variety of sensors that allow robots to see what is happening around them. RGB-D cameras are one of the most popular sensors, due to the richness of the information provided and their reduced price in relation to other types of sensors. On the other side, it is possible to find 2D and 3D LiDARs which can reach a longer sensing distance compared to cameras. Applications can also be found using sonar and other less popular sensors. Section 2 provides details on the characteristics and limitations of these sensors as well as combined approaches such as data fusion.

The second aspect that we consider in this work is *mapping*, since solutions in indoor environments usually make use of a map that facilitates navigation. This is perhaps one of the main differences between outdoor and indoor environments, since indoor environments can be limited to a room, a floor, or an entire building. Outdoor environments, instead, require more extensive plans where care for details is less important. In this field, there is a great diversity of solutions, whose relevance and applicability depend on the application and the specific robot that has to navigate in the target environment. The most relevant types of mapping currently used are presented in Section 3, as well as their limitations and applications.

The third issue that this paper addresses is the *prediction* of trajectories of individuals. While social environments may have a variety of dynamic elements, such as doors and chairs that continuously change their state, people are a crucial factor beyond any object. This is because after correctly identifying an object and its current state, the robot can execute certain behaviors such as evading the object without taking into account further constraints. Instead, for the detection of people, it is not enough to know their current state, but it is necessary to know their future one in the most accurate way, so that the robot not only navigates around them without colliding, but also does it in a socially acceptable manner. This topic will be expanded in Section 4. Moreover, in Section 5, we discuss the corresponding *navigation* methods, where different possible classes of solutions exist. We will focus on predictive and reactive navigation, their distinctive features, as well as their advantages and disadvantages.

Section 6 surveys the most relevant datasets related to applications in the different aspects of social navigation. Furthermore, finally, in Section 7, we present some conclusions based on our study of the subject and future directions regarding the different aspects discussed in this paper. In summary, the contributions of this work are as follows:

- We complete a list of commercial sensors with their characteristics and limitations used in social navigation to ease their selection according to specific application requirements;
- We present different methods for successful navigation, including mapping and trajectory prediction, along with their advantages and limitations, to facilitate their adoption depending on the target scenario;
- We list the available datasets and related work about perception and prediction, which could support the evaluation of social robotics solutions.

## 2. Perception

**Figure 1:**
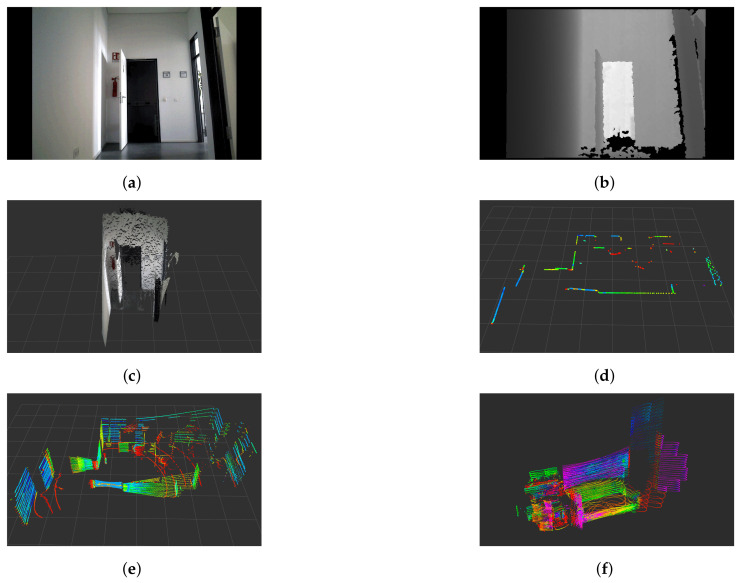
Information given by different types of sensors: (**a**) color image given by the Xtion Pro Live camera; (**b**) depth image given by the Xtion Pro Live camera; (**c**) point-cloud obtained from the color and depth image given by the Xtion Pro Live camera; (**d**) information given by a 2D LiDAR (scan topic from the Velodyne_pointcloud package using a Velodyne VLP-16); (**e**) information given by a 3D LiDAR (point-cloud topic from the Velodyne_pointcloud package using a Velodyne VLP-16); and (**f**) information given by a solid-state LiDAR (Livox Horizon).

As mentioned above, perception is the starting point for building a successful navigation system. However, the possibilities in the selection of one or several sensors are wide, so this section aims to present the most used commercial sensors in service robots, as well as their limitations. The list of sensors presented in this section is based on the product list of some robotics stores such as ROS Components [7] and RobotShop [8]. It is also necessary to clarify that we do not seek to highlight any particular sensor reference, but just show that the selection of the sensors to be used will exclusively depend on the application and its scope. In Figure 1, we present the type of data acquired by different sensors. Figure 1a–c correspond to an RGB-D camera, where is possible to obtain color images, depth images, point-clouds and in specific cases, infrared images. Figure 1d depicts the output obtained with a 2D LiDAR, where a single layer is obtained, in comparison with the result obtained with a conventional 3D LiDAR (Figure 1e), which contains multiple layers from different angles. Finally, Figure 1f shows the data obtained with a solid-state 3D LiDAR.

### 2.1. RGB-D Cameras

Cameras are perhaps the most widely used sensor in robotics regardless of whether it is a social application or not, due to the large amount of information that can be extracted from them. RGB-D cameras provide depth information in addition to color images, which makes them suitable for 3D applications. Table 1 presents a list of RGB-D cameras that we consider common in the social robotic field. Standard RGB cameras are not included in the list due to the great variety of references for this kind of sensor.

Clearly, one of the most relevant features of RGB-D cameras is the color image that other sensors such as LiDARs do not provide. This feature has been exploited in recent years for applications that require the segmentation and detection of objects with high precision, such as those that integrate Convolutional Neural Networks (CNNs) for the detection of faces, human bodies and objects in indoor and outdoor environments such as chairs, doors, walls, floors, cars and trees. Furthermore, for applications that require point-cloud processing, the resolution and frequency that this type of sensor provides are certainly better than those of other sensors. For example, cameras with 640 × 480 pixels of depth resolution can provide up to 307,200 points per sample and 30 samples per second; these numbers could even be improved if a camera with better depth resolution is used. Another important advantage is their price range in relation to the amount of information provided: their average price is lower than those for 2D or 3D LiDARs, and the color and depth information can be obtained at high frequencies.

**Figure 2:**
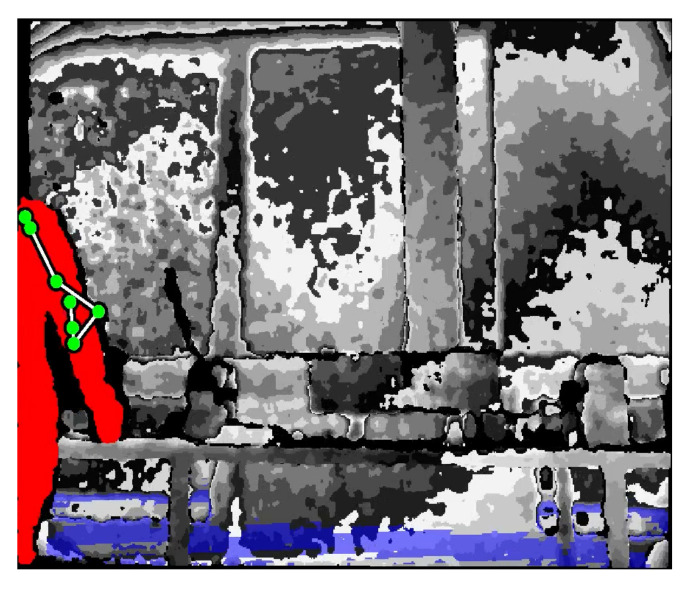
Distorted body detection using the Astra Body Tracker SDK.

Conversely, the field of view (FoV) of cameras, although typically higher in the vertical axis, is more limited in the horizontal axis than that of 2D or 3D LiDARs. Cameras cover at best 110 degrees, while LiDARs can reach up to 360 degrees, covering completely the robot’s surroundings. Another aspect that was a problem until the appearance of references such as ZED and ZED 2 [9] was the detection range, since before these, most cameras did not reach ranges greater than 4 m. Now, products such as the ZED camera can reach ranges of 25 m. Furthermore, depending on the application to be implemented, the FoV could be reduced to a useful area. The restricted FoV, however, could result in issues. For example, the authors in [10] reported distortion at the edge of the image when detecting human body limbs (see Figure 2).

Thanks to the characteristics of this sensor, multiple applications are possible, such as the aforementioned segmentation and detection, which allow robots to have a better understanding of their environment, as well as both geometric and semantic mapping applications. Navigation applications are also possible, but with certain restrictions due to their limited horizontal FoV. For this reason, a 2D or 3D LiDAR is often used in addition. Finally, for robots in social environments, the ability to detect and recognize people using cameras can improve human–robot interaction skills. Nonetheless, applications requiring a high level of privacy may limit or even forbid the use of cameras.

### 2.2. 2D LiDARs

Unlike RGB-D cameras, 2D LiDARs have a wider price range, reaching prices up to thousands of dollars (Table 2). This is due to three general aspects. First, their maximum range, which can vary from 5 m (in the case of the Yujin YRL2-05 [15]) to 100 m or more (in this section, we decided to include the most common references with a range no greater than 25 m, which is enough for most applications in service robotics). The second factor that affects the price is the resolution of the sensor: some of them can provide 1600 points per sample with a separation angle of 0.225 degrees, such as the RPLiDAR A3; others can provide only 360 points per sample with a separation angle of 1 degree, as is the case of the RPLiDAR A1M8 and the Sick TIM551. The third important feature is the frequency of the sensor, which indicates how many samples per second are obtained. For example, in the case of the Hokuyo UST-10LX and UST-20LX, samples are obtained every 0.025 s—while for others such as the RPLiDAR A1M8, samples are obtained approximately every 0.18 s. These three aforementioned characteristics should be considered before acquiring a LiDAR sensor.

One advantage of 2D LiDARs over cameras is their wide horizontal field of view. However, being a 2D sensor with no vertical range, their applications are limited. The most common uses for this type of sensor are mapping, localization and navigation. Nonetheless, unlike 3D sensors such as RGB-D cameras and 3D LiDARs, the amount of information is limited to the extraction of data at the height at which the sensor is located, so certain objects relevant for a given task may not be sensed. Some works have opted for alternatives to create 3D solutions based on 2D LiDARs, for example, by adding an additional degree of freedom to the sensor using a servomotor [16], or by changing the sensor orientation to obtain information on the cross-sectional area of the environment as the robot moves [17]. Additionally, the extraction of information beyond detecting an obstacle is really scarce. An example of this can be the detection of legs with packages such as leg_detector [18], but the accuracy is really low with a large number of false positives, as chair or table legs may be mistakenly detected as human legs.

Due to the limitations of 2D LiDARs, many works have opted for fusing data from different sensors—including by placing a camera in the front part of the robot for semantic information processing in addition to a 2D sensor to consider possible obstacles in the rear part of the robot. Moreover, this type of combination is used to improve the range of camera-based applications. For example, many RGB-D cameras offer a depth range no larger than 4 m, but they could be combined with 2D LiDARs to estimate the position of the person at more than 4 m.

### 2.3. 3D LiDARs

3D LiDARs can be considered intermediate sensors between RGB-D cameras and 2D LiDARs in terms of the information they provide. Unlike 2D LiDARs, they work for applications that require 3D information, but the type of information they gather is not sufficient for tasks such as person recognition. In Table 3, we list some of the 3D LiDARs we consider to be common in robotics. These sensors have a horizontal FoV of 360 degrees and a variable vertical FoV depending on the reference, which is usually smaller than that provided by RGB-D cameras. Normally, this type of sensor reaches large detection distances, so they are commonly used for outdoor applications, more specifically for autonomous driving.

3D mechanical LiDARs have parts that are in motion, so they suffer some wear that decreases their lifetime, unlike cameras. In addition, a distinctive aspect of these sensors is their high price in comparison with others, since they can cost more than USD 1000. However, these prices have been decreasing over time: e.g., the Velodyne LiDAR VLP-16 (now called Velodyne Puck) had a cost of USD 8000 when it was first released, but it can currently be purchased for USD 4000.

A slightly newer alternative to mechanical 3D LiDARs are solid-state LiDARs (Table 4), which limit the use of mechanical parts, achieving greater durability. Additionally, these sensors have better horizontal and vertical resolutions, without, however, reaching the resolution of RGB-D cameras. Furthermore, their frequencies are the same or higher, since they do not have to wait for the mechanism to rotate 360 degrees. Moreover, they have a longer range. However, their FoV is more reduced in some models, e.g., until 81.7° (Livox Horizon) compared to the mechanical LiDARs that can reach until 360°. This characteristic as well as the absence of mechanical parts make their price lower compared to mechanical LiDARs, starting from a few hundred dollars.

Applications such as person recognition are impractical with 3D LiDARs, but it is still possible to detect people as well as certain objects with well-defined physical properties. Indeed, the information provided by these sensors has also been used to train CNNs to improve the accuracy of object detection [19]. Moreover, as with 2D LiDARs, mapping, localization and navigation applications are possible. For the mechanical version, it is possible to perform these tasks without major limitations, while solid-state LiDARs can impose limitations due to their horizontal FoV. Nonetheless, they can still be complemented with other sensors (such as a 2D LiDAR) to offer a better understanding of the environment.

### 2.4. People Detection

In this section, we review the most relevant methods and software packages for people detection using the different sensors presented before.

**2D LiDARs**: As we mentioned above, when this is the only sensor used for detection, methods are constrained to leg or torso detection. In [21], a clustering technique is used to detect pairs of legs with confidence levels to reduce false positives. An approach based on the Fast Fourier Transform has also been presented for legs detection [22]. Moreover, legs can be detected by comparing the sensed data with typical patterns [23], or using machine learning techniques. The authors in [24] used the DROW Laser Dataset to train a CNN with real information, including true and false positive annotations with a certain level of confidence, to improve accuracy. In [25], they detected the torso of human bodies with a Support Vector Data Description. Some software packages (compatible with ROS) available for people detection using 2D LiDARs are the following: [18,26,27,28].

**3D LiDARs**: The authors in [29] detected people in the immediate environment using Online Transfer Learning, by means of CNNs that were trained using information from other sensors such as 2D LiDARs or RGB-D cameras. An online learning framework for human classification using an efficient 3D cluster detector was presented in [30]. In [31], they used a model-based method for people detection, whereby the sensed data are associated with pre-acquired geometrical models that integrate the entire LiDAR scans and model detections using Gaussian shapes. The authors in [32] used segmentation to extract the legs and trunk of human bodies; they matched the two types of detections in order to filter out false positives.

**RGB-D cameras**: The authors in [33] proposed a framework that could detect and track pedestrians in crowded environments. They used the Mask R-CNN, an extension of the Faster R-CNN [34], for the detection step. In [35], they used an upper-body detector to detect people through depth images of up to 5 m (maximal range for the Kinect camera), and a histogram of oriented gradients in specific zones to detect people farther than 5 m. A real-time depth-based template matching people detector was proposed in [36]. In that work, they present different methods to train a depth based-template including multiple upper-body orientations at different height levels, to improve the accuracy. The authors in [37] presented an approach that detects multiple people poses. To correctly detect people, they built a 3D point-cloud from the depth image, clustered all the objects including persons and finally compared it with person and non-person clusters. Additionally, cameras allow us not only to detect people in the environment but also to obtain extra information to improve robot navigation. For example, in [38], they detected and recognized some human activities through the detection of specific body poses, and in [39], they tried to detect some basics emotions.

### 2.5. Summary

Taking into account the advantages and disadvantages of the different kinds of sensors presented, we summarize in Table 5 our recommendation for their selection depending on the application, where ++ shows the sensors that are most easily fit for methods, while − − shows the sensors that are most difficult to fit. These recommendations are further supported throughout the following sections. Regarding 2D mapping, all sensors provide the information necessary to build maps; however, robots with solid-state LiDARs (SSLi) and cameras need to rotate to cover the 360°. To build 3D maps, 2D sensors could be used, but it is necessary to use external hardware to obtain the third dimension; cameras and 3D LiDARs can directly obtain 3D maps. For this reason, we give them the same score: mechanical LiDARs (MLi) can cover 360°, while SSLi and camera reach in the best case 110°; however, the vertical and horizontal resolution of the former could be better in some of the references presented.

For semantic mapping, applications with 2D sensors are really complex because the third dimension is needed. With 3D LiDARs, it is possible to recognize some objects, in particular with SSLi, due to their resolution. However, cameras take the first place in semantic mapping, because their resolution and color images allow robots to implement multiple segmentation methods to recognize more objects in comparison to a LiDAR. Finally, taking into account that the most used method for person recognition is through face or body detection, cameras are the only sensors that can perform this task. However, if the application does not require recognition and detection is enough, there are multiple ways to obtain high accuracy with 3D LiDARs, also providing privacy to the humans involved. Detection applications are also possible with 2D sensors, but with a lower accuracy.

In this section, we presented some popular sensors in social robotics and their characteristics as well as limitations. However, other sensors such as audio systems [40,41] or ultrasonic devices [42] are also used in this field of research and specifically for navigation purposes. Moreover, it is possible to use multiple sensors at the same time (sensor fusion), leveraging the advantages of different devices to improve the results, at the cost of an increase in the computational cost. Finally, we believe that sensors such as mechanical and solid-state LiDARs will continue to reduce their prices, becoming more affordable and more common in this type of applications. Moreover, RGB-D cameras with a better field of view, resolution and depth range should appear in the coming years, allowing to develop new mapping, prediction, and navigation methods in addition to improving the existing ones.

## 3. Mapping

A second aspect to take into account to successfully navigate within an environment is to have a map that contains the necessary information to perform this task. As mentioned in the previous section, each of the techniques described here will have certain advantages and disadvantages that should be taken into account depending on the application. We divided this section in two parts: (1) geometric mapping, where 2D, 2.5D (i.e., 2D maps enhanced with some additional 3D information) and 3D mapping techniques will be presented; (2) semantic mapping, which includes additional information about the objects or people present in the environment. We present geometric mapping as a basis for semantic mapping, as the latter is usually based on 2D/3D standard mapping solutions in which a semantic layer is added to improve the robot behavior. However, it is relevant to emphasize that solutions only based on geometric maps (without semantic information) are insufficient for socially acceptable navigation, which is the focus of this paper.

### 3.1. Geometric Mapping

In this section, we consider geometric maps as those that only include the position of the obstacles in the environment, without differentiating between categories of objects, in order to know whether a robot can or cannot navigate through a specific area. Robot localization and mapping are two problems that are often treated together, as having good localization is necessary to build an accurate map and vice versa [43]. Therefore, we cite some of the main approaches for *Simultaneous Localization and Mapping* (SLAM).

SLAM algorithms are typically based on probabilistic models, using Bayesian inference to solve the problem in two iterative phases: prediction and update. The current position of the robot and the map features are first predicted with some motion model and then updated with the given noisy observations from the available sensors. An option is using a *Kalman Filter* (KF) or its linearized version for non-linear models, the *Extended Kalman Filter* (EKF). Another well-known option is the *Particle Filter* (PF), which uses a set of samples to obtain the distribution of some process giving different weights to each sample. The larger is the weight, the higher the probability is that it is the right solution. There are multiple techniques based on variants of the Particle Filter method, such as FastSlam [44].

#### 3.1.1. 2D SLAM

Although there is a wide variety of 2D SLAM algorithms, we focus here on those most used that offer integration in a robot operating system (ROS). In this list, we find Gmapping [45], HectorSLAM [46], KartoSLAM [47], Google Cartographer [48] and Critical Rays Scan Match (CRSM) SLAM [49]. Other alternative algorithms are CoreSLAM [50], TinySLAM [51] and VinySLAM [52].

The *Gmapping* algorithm is one of the most frequently used in this field, and it is based on the Rao-Blackwellized Particle Filter. This solution assumes that each particle provides an individual map of the environment, and this number of particles is filtered by using the position and movement of the robot as well as the most recent observations. This algorithm is discussed in detail in [53].

**Figure 3:**
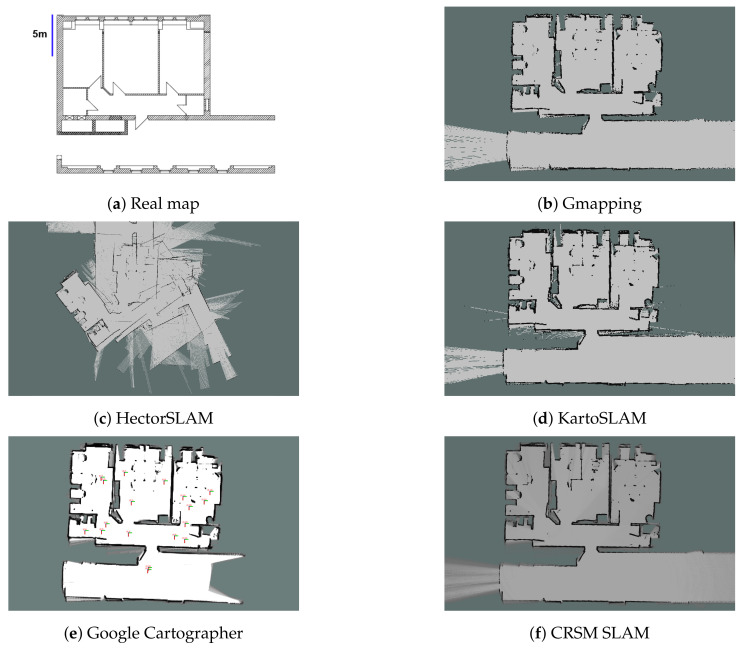
Resulting maps with 2D SLAM algorithms from a pre-recorded dataset [63]. The parameter values are the default ones. The sensor is located at 60 cm of height, has a resolution of 0.4°, a horizontal FoV of 360° and a frequency of 10 Hz.

*HectorSLAM* is another approach for the fast online learning of occupancy grid maps, combining robust laser matching with a system based on inertial sensing [54]. This could be adapted to multiple challenging environments, for example, the authors tested their solution on unmanned ground vehicles in simple environments, as well as environments with complex structures, and it was also additionally tested on an unmanned surface vehicle in a river. This approach works with high-frequency sensors, so if it is used with a sensor whose sampling rate is low, some problems can arise, as shown in Figure 3c. This happens because the approach seeks to match the different samples one behind another, but if the time between samples is big and the robot moves or rotates too quickly, the algorithm cannot match the samples. This method is based on the optimization of beam endpoint alignment using the Gauss–Newton equation.

*KartoSLAM* is a solution based on positional graphs, which means that each node represents the location of the robot along with its sensor data at that position. However, since the processing time may non-linearly increase as the graph becomes larger, this algorithm proposes a method called *Sparse Pose Adjustment*, which efficiently addresses the problem by constructing linear matrices and exploiting their non-iterative Cholesky decomposition [55].

*Google Cartographer* is an algorithm that provides a real-time solution for mapping indoor environments [56]. Unlike most SLAM algorithms, it is not based on particle filters, but it regularly executes a pose optimization to deal with the cumulative error. This algorithm seeks to create sub-maps that are connected by the robot’s position and, when a sub-map is considered complete, it stops processing new information. Its current version performs 2D and 3D mapping, and it allows the configuration of a variety of sensors.

Finally, *CRSM SLAM* uses a scan-to-map matching via a *Random Restart Hill Climbing* algorithm with the intention of reducing noise accumulation, where only critical rays are taken into account for the map update. Moreover, the robot can add new information to the map in the zones already visited, since no part of the map is “closed” until the mapping task is finished by the user [57].

There are works that have sought to compare different SLAM methods, such as [44], where they compare HectorSLAM, Gmapping, KartoSLAM, CoreSLAM and LagoSLAM by presenting the error rate as well as the CPU load of each algorithm. The authors in [58] compared the result of Gmapping, Google Cartographer and HectorSLAM with a ground truth presenting an error table as well as the resulting occupancy grid, showing major drawbacks for HectorSLAM. In [59], Gmapping, HectorSLAM and CRSM SLAM are compared, showing the superiority of the first algorithm with respect to the others. Another recent work [60] compares Gmapping, HectorSLAM, and Karto SLAM, obtaining similar results under specific conditions. Finally, the work presented in [61] compares Gmapping, Google Cartographer and TinySLAM, presenting the root-mean-square error (RMSE) and the resulting map, showing some disadvantages for Cartographer.

We evaluated the five different 2D SLAM methods presented in this section using a laptop with six cores at 2.2 GHz, an i7 processor, and 16 GB of RAM, running Ubuntu 20.04 and ROS Noetic on a Turtlebot 2 with a 2D LiDAR located at 60 cm of height. The sensor has an FoV of 360° and a frequency of 10 Hz. To run the experiment under the same conditions, we used a pre-recorded dataset [62], which contains approximately 380 s of information from a 150 m^2^ indoor environment divided in seven rooms including corridors. The topic used to evaluate this section was /*scan*_*velodyne*, which contains 3805 samples. All methods were executed with default values.

Table 6 shows the comparison of the average processing time spent by the different algorithms to process each sample given by the sensor in the dataset described above. Due to the fact that the different methods update maps at different moments, we mark this time in two different columns; for example, Gmapping updates the map every specific time given by a variable, while CRSM SLAM updates the map every time it processes a laser sample. Regarding the processing time, with the configuration used, we can conclude that KartoSLAM, Google Cartographer and CRSM SLAM more efficiently process the information. Even including the update of the map, the total computation is less than the time in which the sensor delivers a sample (100 ms). For Gmapping or HectorSLAM, updating the map takes more than 100 ms, so it is possible to lose data measurements.

Figure 3 depicts the resulting maps. A great similarity can be observed in most of them except for the one generated by HectorSLAM. This is due to the low frequency of the sensor. Methods such as Google Cartographer and CRSM SLAM, unlike Gmapping and KartoSLAM, do not only use binary values to indicate whether an area is traversable or not, but discrete values between −1 and 100 to create these maps. Thus, it is necessary to consider in more detail the ranges in which an area is or is not traversable when navigating.

#### 3.1.2. 3D SLAM

In terms of 3D SLAM methods, there is a wider variety of approaches. As mentioned above, Google Cartographer is one of the solutions that can also perform 3D mapping with the proper configuration. It is possible to differentiate between solutions specifically developed for RGB-D cameras and others that work with generic point-clouds regardless of the particular sensor.

**Figure 4:**
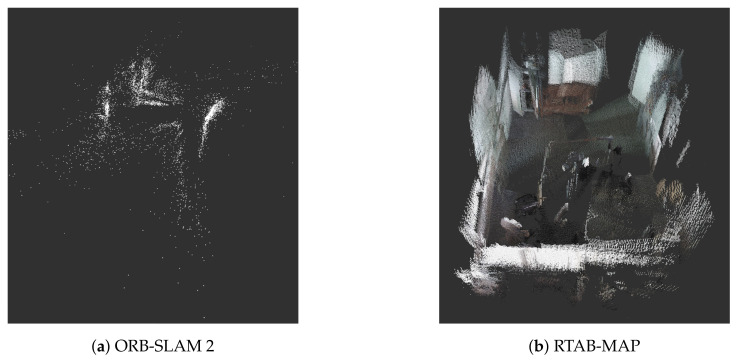
Resulting map for 3D SLAM algorithms using Orbbec Astra Pro camera data from a pre-recorded dataset [63] and the default parameter values (see Table 1 for the camera specifications). The camera was mounted on a Turtlebot 2 at 65 cm of height. The two figures have the same orientation.

In the category of SLAM for cameras, also called visual SLAM, *ORB-SLAM* [64] is a method based on monocular visual features that includes tracking, mapping, relocation and loop closing. Additionally, there exists *ORB-SALM2* [65] which is an extended version that works with stereo and RGB-D cameras in a similar way to the first version. However, this work implements a lighter localization method that reduces the error when closing loops. *RTAB-MAP* [66] is considered a solution similar to Google Cartographer, since it also allows 2D and 3D SLAM through a variety of sensors. It also performs close-loop detection by comparing the current image with those previously stored in the same graph. Each graph is stored in memory and each node is linked to an RGB and depth image. When a loop is closed (i.e., a place that is revisited, closing a part of the map as completed), a graph optimization is executed to correct the poses in the graph. Figure 4 shows a resulting RGB-D map for ORB-SLAM2 in comparison with RTAB-MAP, using a pre-recorded dataset [63] that contains the RGB-D information from an Orbbec Astra Pro camera. The data correspond to an office of approximately 30 m^2^ with the sensor mounted on a Turtlebot 2 at a height of 70 cm; it contains approximately 4400 samples in 150 s. The two images present the same scenario, but it can be seen that the second option presents better results in terms of a more complete view of the environment. This big difference is caused by the fact that ORB-SLAM matches points, while RTAB-MAP matches images. Regarding the processing time, using the same machine of the experiments described in Section 3.1.1, ORB-SLAM2 spends an average time of 0.851 s to update the map, while the RTAB-MAP spends 1.31 s for the complete dataset presented above. A big disadvantage of these methods in comparison with 2D SLAM approaches is that the robot needs to move slowly in order to be able to close the loop timely.

Regarding algorithms that work directly with point-clouds, there are other alternatives. One of the most used SLAM methods with 3D LiDARs is *LiDAR Odometry and Mapping* (LOAM) [16], which divides the problem into two main parts: the first one is to compute the odometry at high frequency to estimate the LiDAR velocity; the second one is in charge of the matching and registration of points at a lower frequency. The authors in [67] proposed a self-supervised LiDAR odometry estimation method in order to improve the final result provided by LOAM. This method applies geometric losses during training by processing the active and previous point nodes. The work in [68] proposes a *Lightweight and Ground-Optimized* (LeGO) LOAM that performs the estimation of the position in real time, as it assumes that the robot moves through a flat ground environment, thus facilitating processing. Segmentation was carried out to find the planar and edge features, and then the algorithm performs an optimization with the Levenberg–Marquardt method. Both works present a comparison between their methods and the original LOAM.

**Figure 5:**
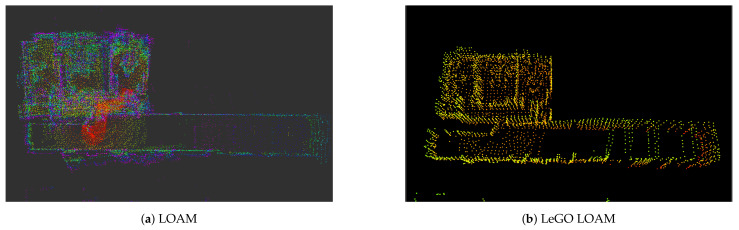
Resulting maps for 3D SLAM algorithms using Velodyne VLP-16 data from a pre-recorded dataset [62] and default parameter values (see Table 3 for LiDAR specifications). The LiDAR was mounted on a Turtlebot 2 at 60 cm of height.

Figure 5 presents the resulting maps for LOAM and LeGO LOAM. We used the same dataset introduced in 2D SLAM [62], but in this case, the topic used was /*velodyne_pointcloud*. In the case of Figure 5a, this method adds all relevant points into the map. However, it can be seen that the map is partially less clear. Figure 5b shows clearer and more ordered map, thanks to the planar and edge detector module. For example, it is possible to distinguish better walls, floors, and roof, and the probability of obtaining a deformed map is lower than with the basic LOAM.

**Figure 6:**
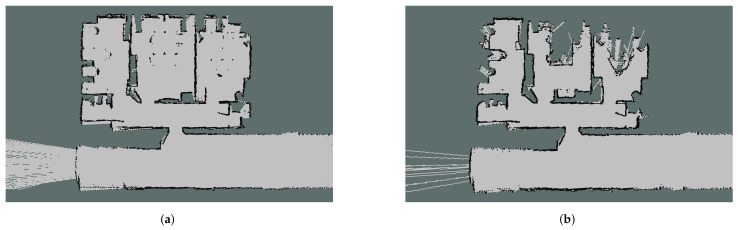
Map comparison between a pure 2D method and a traversable map where the height of the robot restricts the movement in certain areas such as under the tables. The results were obtained from a pre-recorded dataset [63] and default parameter values. The sensor is located at 60 cm of height, the resolution is of 0.4° with an horizontal FoV of 360°, the sensor frequency is 10 Hz and the robot height is 1 m: (**a**) resulting map given by Gmapping with a 2D LiDAR; (**b**) traversable (2.5D) map given by Gmapping using a 3D LiDAR and the Point-Cloud Fast Filter [70].

It should be noted that 2D SLAM algorithms are not limited to 2D sensors and 3D SLAM algorithms are not limited to 3D sensors, because these methods are based on the type of information they receive and not on the specific sensors. In this direction, there are some alternatives, such as using a 2D laser including an additional degree of freedom by driving a servo motor at high frequency in order to obtain information in three dimensions [16]. Another alternative is just changing the default orientation of the sensor to sense traversable sections of the environment to add to the map [17]. Alternatively, 3D sensors can be used with 2D SLAM methods for the creation of 2D maps. For example, the depthimage_to_laserscan package [69], which selects the points at a certain angle to convert the 3D information into 2D; the work presented in [70] also selects the relevant points of the 3D point-cloud and takes into account the height of the robot to build traversable maps, also called 2.5 maps that improve robot 2D navigation. Figure 6 shows a comparison between a pure 2D SLAM approach and a traversable map. It is possible to observe in Figure 6b that certain areas mostly corresponding to tables and chairs are no longer shown as traversable areas for the robot if the robot height is greater than the objects’ height.

### 3.2. Semantic Mapping

When we talk about semantic maps, we refer to the idea of enhancing a geometric map with additional information that may be helpful for robot behavior. For example, detecting structural elements such as doors can help some robots enter or leave a room, or detecting electrical outlets to establish a connection to recharge their own batteries [71]. Furthermore, human detection becomes quite relevant for service robots in social environments, since the way a robot should behave in front of an object is different from the way it should behave in front of a person [72]. Thus, a robot is unable to perform socially acceptable navigation if it does not have the ability to extract basic information from its environment, such as people or special elements detection. This is the main reason to regard semantic mapping as a relevant task in service robot navigation. In this section, we present works related to semantic mapping in relation with social robot navigation.

**Figure 7:**
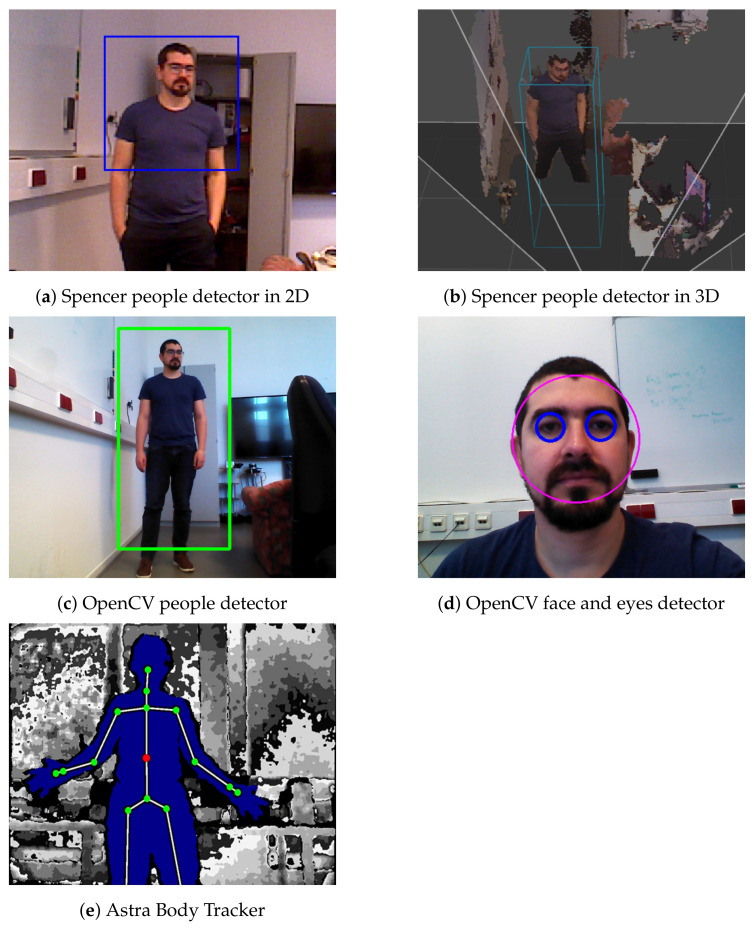
Example of people detection with an RGB-D camera using different approaches.

Cameras are the most used sensors to build semantic maps in indoor environments, as their color images allow them to distinguish between different types of objects and their depth information to locate the elements in a 3-dimensional space. Works in this direction are multiple. For example, the *Astra Body Tracker* (ABT) package [73] uses 2D information from RGB-D cameras to find relevant points on a human body such as the head, shoulders, hands, knees, and feet (Figure 7e). Moreover, CNN-based methods are becoming increasingly popular and used for image segmentation and subsequent object identification. In [74], the authors proposed combining RGB-D SLAM, image-based deep-learning object detection and 3D unsupervised segmentation to create a map that includes not only geometric but also semantic features. Moreover, there are approaches combining CNNs and geometrical-based segmentation [75] or self-supervising training [76]. This method allows users to train the CNN with hundreds or thousands of labeled images that could contain a variety of elements (including humans) and make the detection more accurate.

In [77], they used both a 2D LiDAR and an RGB-D camera to detect people in a crowded environment. They combined an RGB-D upper-body detector, a full-body HOG detector, and an RGB-D detector and the *Point Cloud Library* (i.e., a library for point cloud processing allowing the accurate estimation of people 3D poses). Figure 7a shows detections in an RGB image and Figure 7a shows detections in 3D. Another widespread library including packages for semantic detection and with ROS compatibility is *opencv_apps* [78], which provides functionalities such as people detection (Figure 7c) and face and eyes detection (Figure 7d), among others. All images in Figure 7 were obtained by running the online available versions of the corresponding software packages.

**Figure 8:**
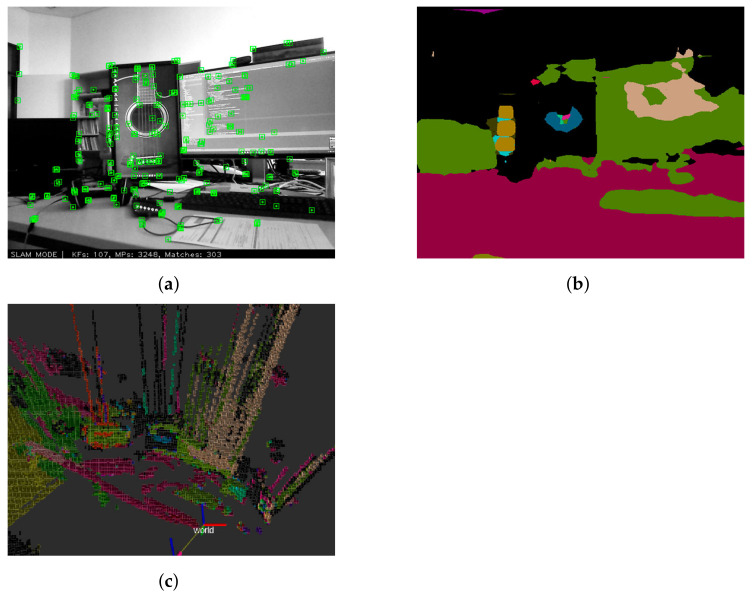
Example of semantic mapping using an RGB-D camera and the Semantic SLAM package [79]: (**a**) camera image with feature extraction; (**b**) Segmented 2D image; (**c**) 3D semantic map.

Other proposals have taken sensing to another level to further improve the interaction of humans and robots within the same environment. For example, by performing the detection of certain human emotions such as happiness, sadness, anger or a neutral state, the robot can behave appropriately to each of these emotions [39]. In Figure 8, we present the result of the *Semantic SLAM* package [79] available online. These algorithms use a pre-trained model to make a segmentation that identifies different types of elements in the environment. They also integrate the previously mentioned ORB-SLAM2 [65] to create a complete semantic 3D map using the colors obtained in the first segmentation and applying them to the resulting 3D point-cloud map.

Additionally, CNN-based techniques can be applied to 3D point-clouds, which enables semantic mapping with 3D LiDARs data. The authors in [80] proposed an architecture called *PointNet*. This method divides its structure into three main parts: the first is a layer that aggregates information from all points; the second is a structure that combines local and global information; finally, two alignment networks align the input data with the characteristics of the points. In [81], *VoxelNet* is presented as a generic 3D detection network that unifies in a single stage the feature extraction and the bounding box prediction. *BirdNet* [82] is another 3D detection framework using LiDAR point-clouds. It is also divided into three stages: the first one projects the information in a cell transforming the LiDAR information into a Beam’s eye view image and normalizing the density of point-clouds in the scene; the second estimates the location using a CNN; finally, a post-processing phase improves the accuracy.

**Figure 9:**
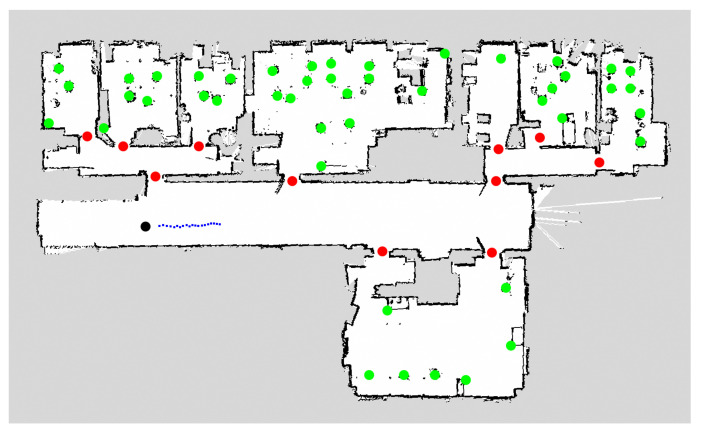
Semantic map obtained with a 3D LiDAR (Velodyne VLP-16) and the approach presented in [32]. Chairs are depicted in green, doors in red and people’s tracked paths in blue. The robot location is given by the black circle.

It is necessary to emphasize that although CNN-based methods can have good accuracy, they require special hardware for training and detection in real time. Moreover, significant effort is needed if there are no datasets for training available. For this reason, applications where it is not necessary to distinguish between all objects in the environment (e.g., the aforementioned ABT) could be used for person detection. Moreover, simple and efficient segmentation methods such as those presented in [32] could be applied. This approach detects objects of simple structures such as doors, chairs, and people (Figure 9), by means of the detection of specific physical features.

### 3.3. Summary

We present different mapping methods such as 2D, 2.5D, 3D and semantic mapping, each with their own characteristics, advantages and disadvantages. All these methods can be helpful depending on the application scenario and requirements. For example, all these approaches could be used for 2D navigation maps, but 2D and 2.5D SLAM techniques would involve less computational effort than the 3D methods. For 3D robot motion, 3D maps (or at least 2.5D) are required. Moreover, in social scenarios, richer and more dynamic maps are needed, which makes semantic mapping more helpful, with people and objects’ information available to improve the robot behavior. Nonetheless, it is necessary to take into account that SLAM remains an open research problem, so we expect new and better methods to appear in following years, improving the current results, and exploiting better hardware to run more powerful methods in real time.

## 4. Human Motion Prediction

In order to apply any method for human trajectory prediction, we assume that fairly robust person detection has been performed, either by means of leg detection, human skeleton detection, CNNs, or another segmentation technique such as those presented in a previous section. In addition, we assume that some method for trajectory tracking has been implemented, since most prediction algorithms base their results on the most recent track of the person, not just the last position.

The most popular method for tracking trajectories in indoor applications is probably the Kalman Filter [21] (and its extended form for non-linear systems [77]) in combination with a data association technique for track matching. Nonetheless, there are different variants. The authors in [83] used another probabilistic method to track multiple persons. Tracking is more stable, as they compute a Jacobian association and the effect of the robot movement to improve its navigation. In [84], the *MDL-tracker*, is proposed, which is based on a bidirectional EKF to compute a set of multiple track hypotheses. The authors in [35] divided the tracking task into two steps: the first one generates candidate trajectories also using a bidirectional EKF, and the second step starts new trajectories to recover possible missing tracks. In general, it should be noted that the achieved accuracy for people detection and tracking is the key to generating good trajectory predictions.

As for trajectory prediction methods, great diversity can be found, from simple algorithms that linearly extend the trajectory of people taking into account the last sensed positions to the detection and learning of patterns using neural networks. Rudenko et al. [85] presented extensive work on this subject, in which they make a classification of the different methods which we adopt in this section. In addition, we discuss in more depth some of the proposals and add more recent publications.

### 4.1. Physics Based

Physics-based prediction methods use one or more physics-based dynamic equations to predict the human motion, and usually use variables such as the person’s current position, last sensed position, linear and angular velocities, and linear and angular accelerations. A major limitation of this type of method is that they do not include information about the environment such as walls or any other obstacles, so the predictions may not be completely accurate. However, these methods are those with less computational requirements in comparison with others.

**Figure 10:**
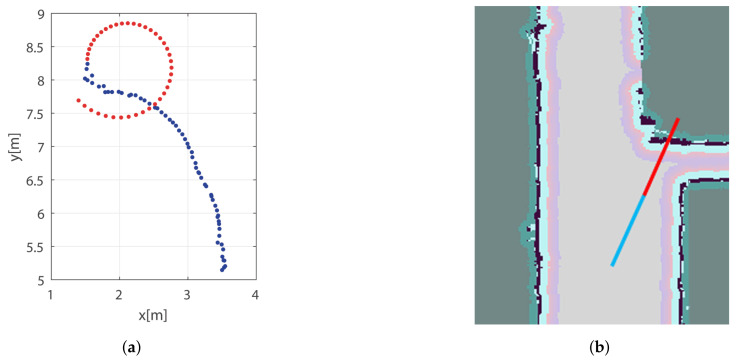
Physics-based methods for human motion prediction: (**a**) trajectory prediction using a CTRA model: sensed positions in blue and predicted positions in red; and (**b**) linearization of trajectory.

An example is the *Constant Turn Rate and Acceleration* (CTRA) model [86] as shown in Figure 10a, where the blue dots show the previously sensed positions and the red dots show the prediction. However, due to the lack of precision in the last sensed position and the angular and linear velocity and acceleration variables, the resulting prediction describes a spiral that is not close to the real trajectory. Another simplified method is the linearization of the trajectories, in order to extend the trajectory based on the last sensed direction and speed of the person. While this approach can be useful in open environments without obstacles or other people, the reality is that most environments are not of this kind. Figure 10b shows the application of this method in a corridor, where the person turns right but the prediction follows a straight line through the wall.

Multi-model solutions are also possible in which, depending on the situation, one or several models are selected to perform the prediction. For example, the authors in [87] formulated the prediction model based on the speed, direction, attraction, penalty, collision and group information of the person. Pellegrini et al. [88] considered a multi-target prediction environment, in which people walk in crowded environments. This work applied a model similar to the previous one, but when two or more predictions cross each other, it changes the model by applying an additional repulsion variable. In [89], a prediction method called *Vulnerable Road Users* is presented. The authors used a *Switching Linear Dynamical System* that integrates multiple motion descriptions into a single model and, depending on the state, it switches to a specific representation. Shi et al. [90] and Huang et al. [91] added a spatial-temporal variable into the model to aggregate information about the social interaction with other persons and capture the multi-modality of motion.

### 4.2. Pattern Based

**Figure 11:**
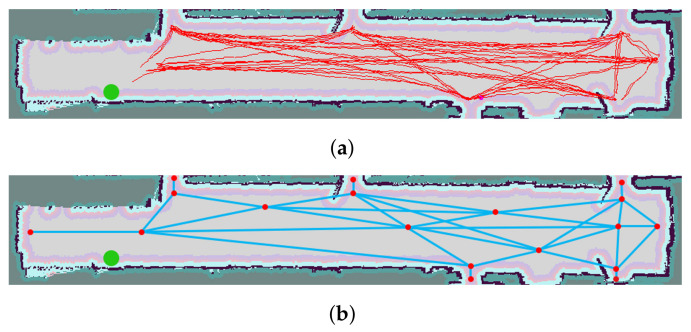
Trajectory patterns extracted in a corridor, taking into account possible destinations. The sensor location is indicated in green: (**a**) Trajectories sensed in the environment; and (**b**) pattern resulting from processing the information.

In the case of pattern-based solutions, dynamic functions learned through training are applied in order to detect patterns that help predict the trajectories of people. Within this type of method, some approaches are based on a single function with no temporal effect, while the so-called sequential methods learn multiple functions to apply depending on the situation and switch between them by using transition functions. In short, these methods use datasets of trajectories to learn patterns, such as those shown in Figure 11a. In the prediction step, once a person is detected, one or multiple dynamical functions are assigned to predict their future path. Another option is to extract the nodes (Figure 11b) that help create patterns, which are then used to predict motion trajectories.

There are multiple examples of sequential methods. Alahi et al. [92] used a *Long-Short Term Memory Network* (LSTM) to learn human motion and predict the future trajectories. This work was applied to crowded scenes in which they take into account a large number of neighbors to estimate trajectories. Xue et al. [93] presented *Social-Scene LSTM*, which also includes the effect of social neighborhood and scene layouts in comparison with the basic LSTM. The method uses three different LSTMs to process person, social, and scene information. Goodhammer et al. [94] proposed a self-learning approach using CNNs with a polynomial approximation. The authors trained a multi-layer perceptron to work with pre-processed trajectories as an input pattern and estimate the future points of the trajectory. The experiments were focused on predictions of up to 2.5 s in the future.

It is also possible to find non-sequential methods, such as that proposed by Xiao et al. [95], in which the authors used a pre-trained *Support Vector Machine* to separate the different tracks and use this information to build trajectory prototypes. These are then used to predict trajectories and match the current stored ones with part of the prototypes, leaving the rest as the predicted trajectory. Luber et al. [96] implemented the theory of learning social-aware *Relative Motion Prototypes*. This method groups similar motion behaviors into clusters that are used to train the network. Moreover, another step is added to include the social context for the prediction by tracking multiple persons. In general, sequential methods present more accurate results than non-sequential methods. This is due to the bigger complexity of their patterns and their adaptability to select the best model to describe the paths, which can cover more possible situations.

### 4.3. Planning Based

Planning-based methods generate hypothetical trajectories based on cost functions that weight the different paths through which people can reach possible destinations within the environment. These methods have been divided into two main approaches: forward planning methods assume the existence of predefined reward functions to compute future trajectories; inverse planning methods do not have predefined reward functions but estimate them by observing datasets of trajectories or through statistical learning. Here, some of the most commons planning algorithms are applied, such as A* or Dijkstra, and more variables could be added to improve the accuracy, such as the head orientation or the tracked path.

**Figure 12:**
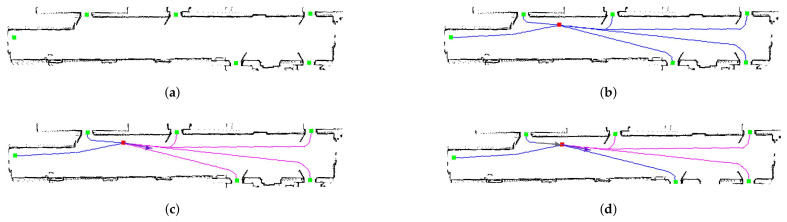
Results for a navigation-based trajectory prediction using the A* algorithm, where the green squares show the possible goals in the environment a people can reach; the red squares show the estimated position of the person sensed; the blue lines show the paths with less probability of being followed; and the magenta lines show the paths with a greater probability of being followed. The gray arrow is the tracked path and the purple arrow is the head orientation. (**a**) Empty environment with possible destinations; (**b**) trajectory prediction using A* algorithm based on the pose estimation and possible goals in the environment. With no variables other than position estimation, all destinations have the same chance of being reached; (**c**) trajectory prediction using A* algorithm based on the pose estimation, possible goals in the environment and head orientation. Taking into account the estimation of the person’s position in the environment and the orientation of the head, it is possible to reduce the destinations that can be reached; (**d**) trajectory prediction using the A* algorithm based on the pose estimation, possible goals in the environment, head orientation and tracked path.

Figure 12 shows the results of planning-based trajectory prediction using the A* algorithm. The method begins with the possible destinations that people can reach in the environment (Figure 12a). From this point, the approach tries to add multiple constraints to improve the cost function and decide which is the most probable trajectory that the person will follow. For example, if only the position of the person is known without other parameters (Figure 12b, in which the person is in red and possible trajectories are in blue), the cost function may assign similar weights to the different options. Another possibility could be using the head orientation (if available) to penalize certain trajectories. In Figure 12c, there are four options with higher probability (magenta) and another two with less probability (blue). Finally, the tracked position of the person could also be used to improve the cost function. In Figure 12c, the last tracked motion is given by the gray arrow. Taking into account this information, only three of the predicted trajectories (magenta) will have a higher probability than the others.

Forward planning methods include solutions such as the one in [97], which present ways to obtain the predicted trajectories through the optimization of the timed elastic band, considering other common factors such as proxemics, group motion and spatial separation between persons. They use local optimization schemes to compute the trajectories. Vasquez [98] identified and addressed three main problems in planning-based methods: their computational cost, their limited ability to model the temporal evolution of the path, and their constant goal assumption. He applied a probabilistic model using parameters such as the velocity, the goal, the velocity model consistency and the cost model consistency, to obtain the predictions. Karasev et al. [99] tried to predict long-term human motion using a jump-Markov process that includes the goal in the model and environmental constraints and biases.

Regarding inverse planning methods, Shen et al. [100] developed transfer learning algorithms that can be trained to estimate cost functions based on the observed trajectory. This work also infers the intentions of the persons that could affect the predictions. Pfeiffer et al. [101] presented an approach based on a feature-based maximum entropy model. They predicted the behavior of heterogeneous groups of people, and their model was trained with human–human interaction information. Chung et al. [102] assumed a social force or spatial effect that forces people to behave in a certain way. The proposed method is based on the *Spatial Behavior Cognition Model*, which includes the feature-based spatial effects.

### 4.4. Summary

In this section, we presented a classification of methods for predicting human trajectories, mentioning some recent and representative works. Each of the explained methods can become as complex as required, including more or less variables within the model. For example, recent works have tried to detect the emotions of people expressed through their facial features [103], so that the robot can behave in an appropriate way according to the detected emotion. Some contributions focus on the prediction of individuals [104], while others include information from groups of persons [105]. Moreover, it is possible to find approaches for open- or close-space environments (a map may be known in the future). However, creating a complete and perfect model for human motion is a tough task. Nonetheless, current models and cost functions could be further improved. We also expect more powerful and accessible processing units that enable human motion prediction in real time.

## 5. Human-Aware Robot Navigation

Navigation is an essential issue in social robotics, since cohabiting with humans in the same environment is not trivial. Robots should behave under certain criteria that allow people to feel comfortable and not be distracted by unusual behaviors within the environment. For this reason, based on semantic mapping and on the prediction of people’s trajectories, valuable works have been published on this topic. Rios et al. [106] presented the relationship and importance of the theory of proxemics in social navigation. Topics such as the classification of the different spaces for individuals as well as for groups are treated, as well as some important parameters related to the robot such as its speed and appearance. This work, unlike the classification we made, classifies the navigation into three groups: *unfocused interaction*, in which the robot is expected to negotiate its position with the people present in the environment through different rules; *focused interaction*, in which the robot is expected to naturally and dynamically adapt to changes; *focused and unfocused interaction*, in which the two previous techniques are combined. Rios et al. identified some challenges in this field, such as the complexity in the management of the different regions addressed by the theory of proxemics and the different factors that can affect these areas, for example, individuals or groups interacting with other people, objects or robots. It also shows the need to extend the understanding and application of the theory of proxemics based on parameters such as the speed and appearance of the robot, among others, and thus dynamically adapt social spaces.

Charalampous et al. [107] also published a review on this topic. Unlike the previous work, this one does not start from the theory of proxemics, but from the mapping stage. The authors divided this topic into three sections: *metric mapping* (traversable or non-traversable areas); *semantic mapping* (detection of objects and people); and *social mapping*, giving more emphasis to the latter. This last type of mapping includes issues such as proxemics and the deformation of social areas depending on certain parameters. It briefly discusses prediction issues, the extraction of information about the human body (speed, orientation, etc.) and from the people context (individuals, groups, interactions, etc.). The authors of this paper recognized the limitations in formulating successful social navigation, such as the modeling and monitoring of the environment and people, extracting more information about human actions and intentions, and how to integrate this into the different layers that contribute to robot control.

Kruse et al. [108] talked about some of the most important challenges such as *comfort* (not disturbing people), *naturalness* (moving in a person-like way) and *sociability* (interaction and prioritization of people when performing actions). On the subject of navigation, the aim is to generalize the steps that must be covered for its execution, which are: the selection of the task (e.g., following a person); defining the target position; defining the path to follow; and finally, adapting the path according to the information perceived by the robot. It also discusses methods based on geometry and machine learning for trajectory prediction. Additionally, the authors note that the accuracy of the prediction considerably decreases if the prediction window is increased, so they consider it important to have a local planner where more variables can be integrated to choose the appropriate behavior for the robot over a shorter time period. An important point that stands out is that of experimentation, since it has certain limitations. For example, in simulation, it is not possible to easily evaluate issues such as comfort and therefore variables of this type cannot be included to modify the path of the robot. Therefore, comfort models must be included in the simulation or experiments must be conducted with real participants who are asked to answer a questionnaire to qualify the robot skills.

Finally, one of the last reviews published on social navigation was by Möller et al. [109], who considered that for a robot to behave in a socially acceptable way, it must have four main features: *active vision*; *navigation*; *robot-human interaction*; and *modeling of human behavior*. In the active vision section, the authors intend to show the need for mobile sensors to acquire more information that can be useful for navigation, as opposed to passive vision (static sensors). In the navigation aspect, they present map-based solutions (e.g., visiting a specific point or area) and those that are not map-based (e.g., exploration). However, they focus on the first type of solution. Being a research work that focuses on the visual part and image-based solutions, a section is added to talk about common learning paradigms for obtaining information that leads to improving the robot’s behavior. In the interaction section, some generalities to take into account are mentioned, to later talk about interaction in industrial contexts and healthcare and assistance at home. In the last section of this work, aspects that must be taken into account to model human behavior such as position estimation and action recognition and how they can be applied to social navigation are presented. One of the major limitations mentioned in this paper is the ability to objectively evaluate socially aware robot navigation.

**Figure 13:**
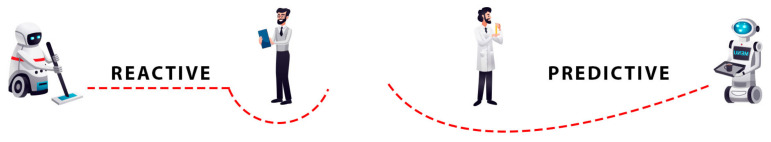
Navigation methods for robot social behavior.

**Figure 14:**
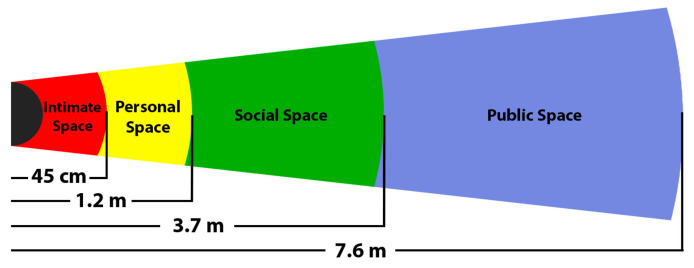
Proxemics theory: different spaces and corresponding distances from a person (in black).

In this work, we classified the contributions in the same way as Cheng et al. [110], who divided these methods into reactive-based planning and predictive-based planning (see Figure 13). Here, we want to mention the importance of the theory of proxemics, which is used to determine which areas around the person are traversable by the robot without causing discomfort. This theory was proposed by Hall [111] and defined four different spaces around the person commonly used in social situations (see Figure 14). The intimate space is usually for friends and family, and it is possible to enter this area by invitation. The personal and social areas are used for common interaction and an “invitation” is not needed. Finally, the public space is an impersonal area that does not affect the person. Moreover, this theory could be useful to distinguish between people and groups of people (the robot’s behavior should be adapted depending on that situation). However, proxemics may also be relaxed to work with emotions and social interactions, e.g., a robot may want to get closer to a happy person than to an angry person [39]. This theory has equally been applied to reactive-based as well as predictive-based navigation.

### 5.1. Reactive-Based Planning

In these methods, the robot changes its direction when a person appears to be blocking its path (Figure 13, left). In short, if it is defined that the robot should move out of the personal space according to the proxemics theory, the robot waits until the distance to the person is approximately 1.2 m and then tries to avoid the person in the best way. In this case, the predicted path of the person is not taken into account. Thus, depending on the speed of the person and the robot, the minimum distance could be increased so that the robot does not evade the person entering their intimate space.

A common approach used in reactive-based navigation is the *Social Force Model*. Tai et al. [112] used this model to define the different forces that are present in the environment (attraction and repulsion) between humans, humans and objects and between objects, and use it as a potential way to navigate the robot towards its goal. Gaussian process regression can also be used to reduce the cost to reach a goal in a dynamic environment. Choi et al. [113] integrated a hierarchical and a non-hierarchical Gaussian process motion controller to carry out collision avoidance, taking into account multiple persons in the scene. Another reactive method successfully applied in the past is *Reciprocal Velocity Obstacle* (RVO), which selects non-intersecting velocities for the persons, considering some reciprocity rules. In [114], a multi-robot navigation approach was proposed, in which RVO is used to model human behavior and replicate it with a robot.

**Figure 15:**
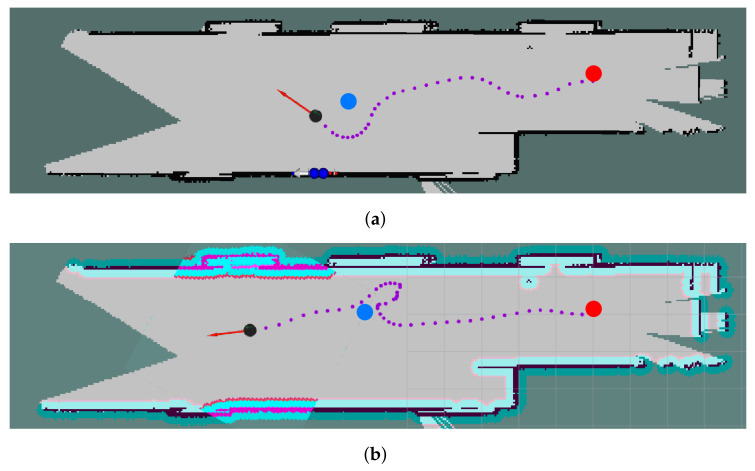
Social navigation examples for the reactive method Sarl* in a corridor scenario. Robot position in black; person position in blue; starting robot position in red; and robot path in purple. In the first situation (**a**) the person was static, while in the second one (**b**) he was walking slowly towards the robot position. In this second case, the robot came closer to the person without respecting the intimate space (Proxemics theory).

Reactive methods are mostly used in unstructured environments, since the prediction of people’s trajectories becomes more complicated when one does not know which possible directions they may take. On the one hand, these methods do not consider people’s future predictions, which could improve the robot behavior to make it more friendly. On the other hand, they are computationally lighter compared to predictive-based methods. As an example of this kind of method, we tested the Sarl* [115] algorithm presented by Li et al., in which social navigation is performed based on a pre-trained model of people’s trajectories. Figure 15 shows the social navigation results of two different experiments in the same environment. The red circle is the starting position of the robot, the blue circle is the detected person, the black circle is the robot current position, and the purple points define the path followed by the robot. We ran this experiment using a Turtlebot 2 with a RPLiDar A2, and the default parameter values for the reactive algorithm. In the first case (Figure 15a), the robot left more free space around the person, trying to avoid them inside their personal space (proxemics theory). In the second case (Figure 15b), the robot temporarily enters the intimate space, due to the time that the controller takes to update the robot trajectory. Moreover, we experienced some limitations in the approach using the default setup. One of them is the people detection range. This is because the algorithm can detect a person between 25 cm and 3 m from the robot position. However, the sensor used (RPLiDAR A2) reaches a detection range of up to 16 m [116]. For this reason, if the robot and a person are moving in opposite directions, they will collide as there is not enough space for stopping or avoiding the person. The second limitation is the robot speed. We used a Turlebot 2 that reaches up to 0.7 m/s. Taking into account that the average human speed is 1 m/s, the robot may not be able to avoid the person in certain situations. The use of a faster robot could improve the performance of the approach.

### 5.2. Predictive-Based Planning

In this type of method, the robot predicts the future states of the people around and then computes its trajectory by taking into account that information to avoid collisions in advance (Figure 13, right). The methods for human motion prediction explained in Section 4 then need to be integrated with the robot navigation. We believe that this type of navigation should take precedence over reaction-based navigation, because if the robot can anticipate people’s behavior in advance, it could adapt its behavior to be more natural and avoid bothering people, in addition to possibly better optimizing resources such as battery and space to travel. Reactive methods should be left for situations in which the robot cannot predict the intentions of surrounding people.

Aoude et al. [117] presented a solution that learns motion patterns by means of Gaussian processes and the RRT-Reach algorithm. The former predicts the state of the dynamic objects and the latter is used as a robust path planner given the uncertain human motion. Lu et al. [118] introduced some social cues, improving the response of the robot when avoiding a person, e.g., in a corridor. They implemented a flexible cost map that re-computes the path every time the map is updated. Vemula et al. [119] presented a framework that models the future trajectories of multiple persons in the environment. They trained a CNN that uses the speed, orientation, and relation with other humans, in order to predict their trajectories and finally used a joint density model that captures the cooperative planning (used for groups) to recompute the trajectory of the robot. CNNs have gained popularity in recent years, including in the robot navigation field. Chen et al. [120] tried to model the human–human, human–robot and crowd–robot interactions to extract some policies that help the robot navigate in crowds. The problem is addressed with one and multiple persons at the scene. Bera et al. [121] presented *SocioSense*, an algorithm for social navigation in real time. They trained a CNN using Bayesian learning and Personality Trait theory, using psychological and social constraints for trajectory planning. This solution was tested in low and medium density crowds. Truong et al. [122] proposed a *Proactive Social Motion Model* (PSMM) that enables the robot to efficiently and safely navigate in crowded environments. This model considers multiple variables of humans in relation to the robot, e.g., position, orientation and speed. It also considers information about human–object and human–group interactions that improve the behavior of the robot. PSMM implements a variant of the social force model and a hybrid Reciprocal Velocity Obstacle technique.

Predictive-based planning approaches depend on how robots predict the future states of the people on the scene, and on how they plan their actions according to these predictions. These navigation approaches could be simpler considering a single human or more complex with multi-human scenarios, and even non-structured environments. The results found in the literature for predictive approaches are generally better than reactive ones, especially in large and cluttered environments, due to the additional information exploited in relation to human motion prediction. However, these methods also need more computation resources to be trained as well as to be executed in real time.

### 5.3. Summary

Both types of planning methods, reactive and predictive, have advantages and disadvantages. For example, reactive-based methods are usually easier and faster to implement due their complexity in relation to predictive-based methods. On the other hand, the predictive-based methods have a more acceptable behavior in social environments, and robots could avoid persons in a more comfortable way, without disturbing persons by entering in their personal space. However, in a social environment, it is common to find situations in which the robot cannot avoid a person using a predictive-based method, e.g., in the case of a person coming out of a room that the robot is supposed to enter. Moreover, we consider that the robot’s maximal speed is an important factor in social navigation, because depending on this variable, the robot could or could not follow some social rules. Another limitation that makes it difficult to compare the different approaches is the lack of well-established benchmarks for social robot navigation.

## 6. Related Datasets

Table 7 lists some of the most relevant datasets to test different methods related to social navigation. This table also includes the type of information provided by sensors, either images or point-clouds (depending on the sensor). It is also indicated whether each dataset includes pedestrian annotations. Further discussion of those datasets can be seen in [123]. The *Newer College Dataset* (NCD) contains information from an Intel Realsense D435i (RGB-D camera) and an Ouster OS-1 64 (3D mechanical LiDAR) recorded in an outdoor environment. The *Kitti* dataset is a popular set of information with camera and LiDAR information, which is especially used to test self-driving car systems. The recently created Stanford Drone Dataset (SDD) contains multiple image sequences including bicyclists, skateboarders, cars, buses, and golf carts. Even though the dataset is related to drone applications, it can also be useful for people detection systems with top-view cameras. In addition, the ETH and UCY datasets contain multiple sequences of videos that are used for person detection and tracking. These datasets are some of the most used ones to test and compare results in person motion prediction. Finally, the recent JRBD dataset provides 2D and 3D information with more than 2 million annotated boxes and trajectories that allow training algorithms for human detection and motion prediction. The nuScene is another outdoor dataset recorded for self-driving solutions, but it contains useful information for object detection and tracking (including people). This dataset contains 1000 scenes with camera, 3D LiDARs and sonar information.

## 7. Conclusions and Future Work

We presented relevant topics in the field of social robotics, especially those involved in social navigation for service robots. In this sense, we mentioned some representative works in the different related sub-areas and their review.

Nowadays, we have a variety of sensors that allow robots to perceive their surroundings, and the related technologies are continuously advancing. We observed that RGB-D cameras are the most popular sensors due the richer information they provide and their fairly reasonable price. However, other sensors such as mechanical 3D LiDARs or solid state LiDARs are also improving their performance and decreasing their prices. For this reason, we believe that applications based on LiDARs will become quite popular in the coming years. However, we also consider that applications related with the recognition of people’s activities and intentions, which are clearly simpler using RGB-D cameras, will be of interest in the near future, as they can allow social robots to adapt to a larger number of settings.

Regarding the SLAM problem, we show the results of some common approaches and describe their advantages and disadvantages, in order to facilitate the task of selecting a proper solution for each application. In the results presented in the section related to 2D SLAM, we see that the maps built by the different algorithms are similar under the situation described except for HectorSLAM. However, the issue with this approach can be easily solved using a sensor with higher frequency. Moreover, we observed that almost all methods build static maps. For this reason, if something changes in the environment, the map needs to be built again. In this case, algorithms that can handle updates in the environment could be useful. On the other side, due the complexity of the situations that must be handled, semantic maps are really useful. However, almost all methods require special hardware to be executed in real time without losing sensor information. For this reason, it is necessary to develop more efficient ways to detect the different objects that could affect the navigation of the robot in social environments. Moreover, approaches for active robot vision [109] may lead to a better understanding of the scene, by using mobile sensors instead of the traditional static views.

Human motion prediction is also a trending topic, as more and more robots are being deployed in environments with humans. There exist complex solutions that predict trajectories for groups in crowded environments, and some of them even detect human emotions and exploit their impact on human motion to improve the prediction. Furthermore, more complex models for predicting human trajectories have been developed to include more variables, not only focusing on individuals but also on groups of people, activities, intentions, human–robot interaction, etc. However, this kind of solution becomes more difficult to apply and requires more processing resources. Finally, it is necessary to clarify that a degree of uncertainty in people’s decisions is always present and cannot be accurately predicted, thus affecting the resulting accuracy.

Finally, social navigation is a topic that directly depends on how robots perceive dynamic objects and estimate their future states. As we present in this work, both predictive- and reactive-based methods have their own advantages and disadvantages. In terms of efficiency, predictive-based methods are better due to their path optimization. However, reactive-based methods are easier to apply given that it is not necessary to predict the path of persons and recompute the robot path in advance. Moreover, we believe that social environments present multiple and heterogeneous situations in which each of these methods manifests specific trade-offs. For this reason, a solution that integrates the different methods could provide more flexibility and offer better results in general. In addition, one of the most important aspects to be defined in social navigation is objective metrics to evaluate the different methods. Those typically used, such as comfort, naturalness and sociability, present a high degree of complexity, since they are measured through personal surveys that tend to be subjective.

## Figures and Tables

**Table 1:** The relevant characteristics of cameras used in service robots. RGB and depth resolution working at 30 fps. FoV values correspond to the depth image. The Intel D435i and D455 include an IMU. Elements with (*) are currently discontinued.

Reference	FoV (H × V)	Depth Range (m)	RGB Resolution	Depth Resolution	Price
Orbbec Astra Pro [11]	60° × 49.5°	0.4–8	1280 × 720	640 × 480	USD 149.99
Orbbec Persee [11]	60° × 49°	0.6–8	1280 × 720	640 × 480	USD 239.99
RealSense D435i [12]	87° × 58°	0.3–3	1920 × 1080	1280 × 720	USD 209
RealSense D455 [12]	87° × 58°	0.6–6	1280 × 800	1280 × 720	USD 249
× tion Pro Live [13]	58° × 45°	0.8–3.5	1280 × 1024	640 × 480	USD 300 *
× tion 2 [13]	74° × 52°	0.8–3.5	2592 × 1944	640 × 480	USD 430
Azure Kinect DK [14]	75° × 65°	0.5–3.86	3840 × 2160	640 × 576	USD 399
ZED [9]	90° × 60°	0.5–25	1920 × 1080	1920 × 1080	USD 349
ZED 2 [9]	110° × 70°	0.2–20	1920 × 1080	1920 × 1080	USD 449

**Table 2:** Relevant characteristics of 2D LiDARs used for perception in service robots.

Reference	FoV	Range (m)	Resolution	Frecuency	Price
Hokuyo UST-10LX [7]	270°	0.06–10	0.25°	40 Hz	USD 1600
Hokuyo URG-04LX [7]	240°	0.06–4.095	0.36°	10 Hz	USD 2100
Hokuyo UST-20LX [7]	270ΰ	0.06–20	0.25ΰ	40 Hz	USD 2700
Sick TIM551 [7]	270°	0.05–10	1°	15 Hz	USD 1880
Sick TIM571 [7]	270°	0.05–25	0.33°	15 Hz	USD 1623
RPLIDAR A1M8 [8]	360°	0.15–12	1°	5.5 Hz	USD 330
RPLIDAR A2M8 [7]	360°	0.15–6	0.9°	10 Hz	USD 389
RPLIDAR A2M6 [7]	360°	0.2–18	0.45°	15 Hz	USD 636
RPLIDAR A3 [7]	360°	0.2–25	0.225°	15 Hz	USD 549
Yujin YRL2-05 [8]	270°	0.1–5	0.55°	20 Hz	USD 650

**Table 3:** Relevant characteristics of mechanical 3D LiDARs used for robot perception.

Reference	FoV H × V	Range (m)	Resolution H × V	Frequency	Price
RS-LiDAR-16 [8]	360° × 30°	150	0.2° × 2°	10 Hz	USD 5312
OLEI LR-16F [8]	360° × 30°	100	0.18° × 2°	10 Hz	USD 1750
LS 32 Channel [8]	360° × 30°	150	0.18° × 1°	10 Hz	USD 5000
M8-1 Plus LIDAR [8]	360° × 20°	150	0.132° × 2.85°	10 Hz	USD 5007
Velodyne Puck	360° × 30°	100	0.2° × 2°	10 Hz	USD 4000

**Table 4:** Relevant characteristics of solid-state 3D LiDARs used for robot perception.

Reference	FoV H × V	Range (m)	Resolution H × V	Frequency	Price
Livo × Mid-40 [20]	38.4° circular	260	0.03° × 0.28°	N/A	USD 599
Livo × Horizon [20]	81.7° × 25.1°	260	0.3° × 0.28°	N/A	USD 800
Livo × TELE-15 [20]	14.5° × 16.2°	500	0.02° × 0.12°	10 Hz	USD 1200
CE30-D [8]	60° × 40°	150	N/A × 2°	30 Hz	USD 1199
CE30-A [8]	132° × 9°	4	N/A	20 Hz	USD 650

**Table 5:** Recommendations for sensor selection.

Sensor	2D Mapping	3D Mapping	Semantic Mapping	People Detection	People Recognition
2D LiDARs	+ +	− −	− −	−	− −
3D MLi	+ +	+ +	+	+ +	− −
3D SSLi	+	+ +	+	+ +	− −
RGB-D cameras	+	+ +	+ +	+ +	+ +

**Table 6:** The processing time of 2D SLAM algorithms on a pre-recorded dataset [62] and with the default parameter values. Sensor height: 60 cm; resolution: 0.4°; horizontal FoV: 360°; and frequency: 10 Hz.

Method	Processing Time (without Map Update)	Processing Time (with Map Update)
Gmapping	0.192 ms	2137.34 ms
HectorSLAM	79 ms	125.84 ms
KartoSLAM	0.387 ms	52.87 ms
Google Cartographer	0.231 ms	12.43 ms
CRSM SLAM	97 ms	97 ms

**Table 7:** Relevant datasets to test methods related to social navigation.

Name			Data			Related Work
			Point-Cloud			
	RGB Images	Camera	2D LiDAR	3DLiDAR	Annotations	
NCD [124]	✓	x	x	✓	x	**LiDAR-based SLAM**: [125,126,127,128,129,130]
KITTI [131]	✓	x	x	✓	✓	**LiDAR-based SLAM:** [124,126,132,133,134] **Visual SLAM**: [135,136,137,138,139]
SDD [105]	✓	x	x	x	✓	**People detection**: [140,141,142,143,144] **Trajectory prediction**: [145,146,147,148,149,150,151,152,153,154,155,156]
ETH [157]	✓	x	x	x	✓	**People detection**: [36] **Trajectory prediction**: [93,119,145,146,150,151,158,159,160]
UCY	✓	x	x	x	✓	**Trajectory prediction**: [93,145,146,150,151,158,159,160]
JRDB [105]	✓	x	x	✓	✓	**People detection**: [161,162,163,164,165] **Social navigation**: [166]
nuScenes [167]	✓	x	x	✓	✓	**People detection**: [168,169,170,171,172] **Trajectory prediction**: [173,174,175]

## Abbreviations

The following abbreviations are used in this manuscript:

FoV	Field of View
SSLi	Solid-State LiDARs
MLi	Mechanical LiDARs
LOAM	LiDAR Odometry and Mapping
CNN	Convolutional Neural Networks
KF	Kalman Filter
EKF	Extended Kalman Filter
PF	Particle Filter
ROS	Robot Operating System
SLAM	Simultaneous Localization and Mapping
SDD	Stanford Drone Dataset
PSMM	Proactive Social Motion Model

## References

[1] Perez-Higueras N., Ramon-Vigo R., Perez-Hurtado I., Capitan J., Caballero F., Merino L. A social navigation system in telepresence robots for elderly. Proceedings of the Workshop on the International Conference on Social Robotics.

[2] Belpaeme T., Kennedy J., Ramachandran A., Scassellati B., Tanaka F. (2018). Social robots for education: A review. Sci. Robot..

[3] Triebel R., Arras K., Alami R., Beyer L., Breuers S., Chatila R., Chetouani M., Cremers D., Evers V., Fiore M. (2016). Spencer: A socially aware service robot for passenger guidance and help in busy airports. Field and Service Robotics.

[4] Pérez-Higueras N., Ramón-Vigo R., Caballero F., Merino L. Robot local navigation with learned social cost functions. Proceedings of the 11th International Conference on Informatics in Control, Automation and Robotics (ICINCO).

[5] Biswas J., Veloso M.M. (2013). Localization and navigation of the cobots over long-term deployments. Int. J. Robot. Res..

[6] Irobot Roomba Vacuum Cleaner. https://www.irobot.de/.

[7] ROS Components Robotics Store. https://www.roscomponents.com/en/.

[8] Robot Shop Robotics Store. https://www.robotshop.com/en/.

[9] Stereolabs Zed Cameras. https://www.stereolabs.com/.

[10] Janzon S., Sánchez C.M., Zella M., Marrón P.J. Person Re-Identification in Human Following Scenarios: An Experience with RGB-D Cameras. Proceedings of the Fourth IEEE International Conference on Robotic Computing (IRC).

[11] ORBBEC Astra Cameras. https://orbbec3d.com/.

[12] Intel RealSense Cameras. https://store.intelrealsense.com/.

[13] Xtionprolive Xtion Cameras. http://xtionprolive.com/.

[14] Microsoft Azure Kinect DK. https://www.microsoft.com/en-us/p/azure-kinect-dk/8pp5vxmd9nhq?activetab=pivot:techspecstab.

[15] Yujinrobot YRL2-05. https://yujinrobot.com/autonomous-mobility-solutions/components/lidar/.

[16] Zhang J., Singh S. LOAM: Lidar Odometry and Mapping in Real-time. Proceedings of the Robotics: Science and Systems.

[17] Chen M., Yang S., Yi X., Wu D. Real-time 3D mapping using a 2D laser scanner and IMU-aided visual SLAM. Proceedings of the IEEE International Conference on Real-time Computing and Robotics (RCAR).

[18] Pantofaru C. Leg Detector. http://wiki.ros.org/leg_detector.

[19] Sun R., Gao Y., Fang Z., Wang A., Zhong C. (2019). Ssl-net: Point-cloud generation network with self-supervised learning. IEEE Access.

[20] Livox LiDARs Store. https://www.livoxtech.com/.

[21] Leigh A., Pineau J., Olmedo N., Zhang H. Person tracking and following with 2d laser scanners. Proceedings of the 2015 IEEE International Conference on Robotics and Automation (ICRA).

[22] Bayu Dewantara B.S., Dhuha S., Marta B.S., Pramadihanto D. FFT-based Human Detection using 1-D Laser Range Data. Proceedings of the 2020 International Seminar on Intelligent Technology and Its Applications (ISITIA).

[23] Bellotto N., Hu H. (2008). Multisensor-based human detection and tracking for mobile service robots. IEEE Trans. Syst. Man Cybern. Part B Cybern..

[24] Beyer L., Hermans A., Linder T., Arras K.O., Leibe B. (2018). Deep person detection in 2D range data. arXiv.

[25] Jung E.J., Lee J.H., Yi B.J., Park J., Yuta S., Noh S.T. (2014). Development of a Laser-Range-Finder-Based Human Tracking and Control Algorithm for a Marathoner Service Robot. IEEE/ASME Trans. Mechatron..

[26] Pantofaru C., Meyer O. Cob Leg Detector. http://wiki.ros.org/cob_leg_detection.

[27] Leigh A., Pineau J., Olmedo N., Zhang H. Leg Tracker. https://github.com/angusleigh/leg_tracker.

[28] Bellotto N., Hu H. Edge Leg Detector. https://github.com/marcobecerrap/edge_leg_detector.

[29] Yan Z., Sun L., Duckctr T., Bellotto N. Multisensor Online Transfer Learning for 3D LiDAR-Based Human Detection with a Mobile Robot. Proceedings of the 2018 IEEE/RSJ International Conference on Intelligent Robots and Systems (IROS).

[30] Yan Z., Duckett T., Bellotto N. Online learning for human classification in 3D LiDAR-based tracking. Proceedings of the 2017 IEEE/RSJ International Conference on Intelligent Robots and Systems (IROS).

[31] Zhang S., Wang D., Ma F., Qin C., Chen Z., Liu M. Robust Pedestrian Tracking in Crowd Scenarios Using an Adaptive GMM-based Framework. Proceedings of the 2020 IEEE/RSJ International Conference on Intelligent Robots and Systems (IROS).

[32] Sánchez C.M., Zella M., Capitán J., Marrón P.J. (2020). Semantic Mapping with Low-Density Point-Clouds for Service Robots in Indoor Environments. Appl. Sci..

[33] Chandra R., Bhattacharya U., Bera A., Manocha D. DensePeds: Pedestrian Tracking in Dense Crowds Using Front-RVO and Sparse Features. Proceedings of the 2019 IEEE/RSJ International Conference on Intelligent Robots and Systems (IROS).

[34] Jiang H., Learned-Miller E. Face detection with the faster R-CNN. Proceedings of the 2017 12th IEEE International Conference on Automatic Face & Gesture Recognition (FG 2017).

[35] Jafari O.H., Mitzel D., Leibe B. Real-time RGB-D based people detection and tracking for mobile robots and head-worn cameras. Proceedings of the IEEE International Conference on Robotics and Automation (ICRA).

[36] Jafari O.H., Yang M.Y. Real-time RGB-D based template matching pedestrian detection. Proceedings of the 2016 IEEE International Conference on Robotics and Automation (ICRA).

[37] Lewandowski B., Liebner J., Wengefeld T., Müller S., Gross H.M. Fast and Robust 3D Person Detector and Posture Estimator for Mobile Robotic Applications. Proceedings of the 2019 International Conference on Robotics and Automation (ICRA).

[38] Sung J., Ponce C., Selman B., Saxena A. Unstructured human activity detection from RGBD images. Proceedings of the 2012 IEEE International Conference on Robotics and Automation.

[39] Narayanan V., Manoghar B.M., Sashank Dorbala V., Manocha D., Bera A. ProxEmo: Gait-based Emotion Learning and Multi-view Proxemic Fusion for Socially-Aware Robot Navigation. Proceedings of the IEEE/RSJ International Conference on Intelligent Robots and Systems (IROS).

[40] Pleshkova S., Zahariev Z. Development of system model for audio visual control of mobile robots with voice and gesture commands. Proceedings of the 40th International Spring Seminar on Electronics Technology (ISSE).

[41] Telembici T., Grama L., Rusu C. Integrating Service Robots into Everyday Life Based on Audio Capabilities. Proceedings of the International Symposium on Electronics and Telecommunications (ISETC).

[42] Lim J., Lee S., Tewolde G., Kwon J. Indoor localization and navigation for a mobile robot equipped with rotating ultrasonic sensors using a smartphone as the robot’s brain. Proceedings of the IEEE International Conference on Electro/Information Technology (EIT).

[43] Fuentes-Pacheco J., Ruiz-Ascencio J., Rendón-Mancha J.M. (2015). Visual simultaneous localization and mapping: A survey. Artif. Intell. Rev..

[44] Santos J.M., Portugal D., Rocha R.P. An evaluation of 2D SLAM techniques available in robot operating system. Proceedings of the IEEE International Symposium on Safety, Security, and Rescue Robotics (SSRR).

[45] Gerkey B. Gmapping Algorithm. http://wiki.ros.org/gmapping.

[46] Kohlbrecher S., Meyer J. HectorSLAM Algorithm. http://wiki.ros.org/hector_slam.

[47] Gerkey B. KartoSLAM Algorithm. http://wiki.ros.org/slam_karto.

[48] The Cartographer Authors GoogleCartographer Algorithm. https://google-cartographer-ros.readthedocs.io/en/latest/.

[49] Tsardoulias M. CRSMSLAM Algorithm. http://wiki.ros.org/crsm_slam.

[50] Steuxa B., El Hamzaoui O. CoreSLAM Algorithm. http://library.isr.ist.utl.pt/docs/roswiki/coreslam.html.

[51] JetBrains Research, Mobile Robot Algorithms Laboratory TinySLAM Algorithm. https://github.com/OSLL/tiny-slam-ros-cpp.

[52] JetBrains Research, OSLL Team VinySLAM Algorithm. http://wiki.ros.org/slam_constructor.

[53] Grisetti G., Stachniss C., Burgard W. (2007). Improved techniques for grid mapping with rao-blackwellized particle filters. IEEE Trans. Robot..

[54] Kohlbrecher S., Von Stryk O., Meyer J., Klingauf U. A flexible and scalable SLAM system with full 3D motion estimation. Proceedings of the IEEE International Symposium on Safety, Security, and Rescue Robotics.

[55] Konolige K., Grisetti G., Kümmerle R., Burgard W., Limketkai B., Vincent R. Efficient sparse pose adjustment for 2D mapping. Proceedings of the IEEE/RSJ International Conference on Intelligent Robots and Systems.

[56] Hess W., Kohler D., Rapp H., Andor D. Real-time loop closure in 2D LIDAR SLAM. Proceedings of the IEEE International Conference on Robotics and Automation (ICRA).

[57] Tsardoulias E., Petrou L. (2013). Critical rays scan match SLAM. J. Intell. Robot. Syst..

[58] Yagfarov R., Ivanou M., Afanasyev I. Map comparison of lidar-based 2D slam algorithms using precise ground truth. Proceedings of the 15th International Conference on Control Automation, Robotics and Vision (ICARCV).

[59] Rojas-Fernández M., Mújica-Vargas D., Matuz-Cruz M., López-Borreguero D. Performance comparison of 2D SLAM techniques available in ROS using a differential drive robot. Proceedings of the International Conference on Electronics, Communications and Computers (CONIELECOMP).

[60] Xuexi Z., Guokun L., Genping F., Dongliang X., Shiliu L. SLAM algorithm analysis of mobile robot based on lidar. Proceedings of the Chinese Control Conference (CCC).

[61] Krinkin K., Filatov A., Filatov A.Y., Huletski A., Kartashov D. Evaluation of Modern Laser Based Indoor SLAM Algorithms. Proceedings of the 22nd Conference of Open Innovations Association (FRUCT).

[62] Medina C. Rosbag Dataset for 2D SLAM. To Use This Dataset Cite This Work. https://drive.google.com/file/d/1-ADUs3CD1qgrY8bW3LF9TOUDvPTtjc8-/view?usp=sharing.

[63] Medina C. Rosbag Dataset with RGB-D Camera Data for 3D SLAM. To Use This Dataset Cite This Work. https://drive.google.com/file/d/15Ew5OEN5oJwmfQLKiepka1cVhMxNgaay/view?usp=sharing.

[64] Mur-Artal R., Montiel J.M.M., Tardós J.D. (2015). ORB-SLAM: A Versatile and Accurate Monocular SLAM System. IEEE Trans. Robot..

[65] Mur-Artal R., Tardós J.D. (2017). ORB-SLAM2: An Open-Source SLAM System for Monocular, Stereo, and RGB-D Cameras. IEEE Trans. Robot..

[66] Labbé M., Michaud F. (2019). RTAB-Map as an open-source lidar and visual simultaneous localization and mapping library for large-scale and long-term online operation. J. Field Robot..

[67] Nubert J., Khattak S., Hutter M. (2020). Self-supervised Learning of LiDAR Odometry for Robotic Applications. arXiv.

[68] Shan T., Englot B. Lego-loam: Lightweight and ground-optimized lidar odometry and mapping on variable terrain. Proceedings of the IEEE/RSJ International Conference on Intelligent Robots and Systems (IROS).

[69] Rockey C. Depth Image to LaserScan. http://wiki.ros.org/depthimage_to_laserscan.

[70] Sánchez C.M., Zella M., Capitan J., Marron P.J. Efficient Traversability Mapping for Service Robots Using a Point-cloud Fast Filter. Proceedings of the 19th International Conference on Advanced Robotics (ICAR).

[71] Meeussen W., Wise M., Glaser S., Chitta S., McGann C., Mihelich P., Marder-Eppstein E., Muja M., Eruhimov V., Foote T. Autonomous door opening and plugging in with a personal robot. Proceedings of the IEEE International Conference on Robotics and Automation.

[72] Bhagya S.M., Samarakoon P., Chapa Sirithunge H.P., Muthugala M.A.V.J., Muthugala J., Buddhika A.G., Jayasekara P. Proxemics and Approach Evaluation by Service Robot Based on User Behavior in Domestic Environment. Proceedings of the IEEE/RSJ International Conference on Intelligent Robots and Systems (IROS).

[73] Astra Body Tracker. https://github.com/KrisPiters/astra_body_tracker.

[74] Sünderhauf N., Pham T.T., Latif Y., Milford M., Reid I. Meaningful maps with object-oriented semantic mapping. Proceedings of the IEEE/RSJ International Conference on Intelligent Robots and Systems (IROS).

[75] Nakajima Y., Tateno K., Tombari F., Saito H. Fast and accurate semantic mapping through geometric-based incremental segmentation. Proceedings of the IEEE/RSJ International Conference on Intelligent Robots and Systems (IROS).

[76] Ma L., Stückler J., Kerl C., Cremers D. Multi-view deep learning for consistent semantic mapping with rgb-d cameras. Proceedings of the IEEE/RSJ International Conference on Intelligent Robots and Systems (IROS).

[77] Linder T., Breuers S., Leibe B., Arras K.O. On multi-modal people tracking from mobile platforms in very crowded and dynamic environments. Proceedings of the IEEE International Conference on Robotics and Automation (ICRA).

[78] OpenCV Apps for ROS. http://wiki.ros.org/opencv_apps.

[79] Xuan Z., David F. (2018). Real-Time Voxel Based 3D Semantic Mapping with a Hand Held RGB-D Camera. https://github.com/floatlazer/semantic_slam.

[80] Qi C.R., Su H., Mo K., Guibas L.J. Pointnet: Deep learning on point sets for 3D classification and segmentation. Proceedings of the IEEE Conference on Computer Vision and Pattern Recognition.

[81] Zhou Y., Tuzel O. Voxelnet: End-to-end learning for point cloud based 3D object detection. Proceedings of the IEEE Conference on Computer Vision and Pattern Recognition.

[82] Beltrán J., Guindel C., Moreno F.M., Cruzado D., Garcia F., De La Escalera A. Birdnet: A 3D object detection framework from lidar information. Proceedings of the 21st International Conference on Intelligent Transportation Systems (ITSC).

[83] Ban Y., Alameda-Pineda X., Badeig F., Ba S., Horaud R. Tracking a varying number of people with a visually-controlled robotic head. Proceedings of the IEEE/RSJ International Conference on Intelligent Robots and Systems (IROS).

[84] Breuers S., Beyer L., Rafi U., Leibel B. Detection- Tracking for Efficient Person Analysis: The DetTA Pipeline. Proceedings of the IEEE/RSJ International Conference on Intelligent Robots and Systems (IROS).

[85] Rudenko A., Palmieri L., Herman M., Kitani K.M., Gavrila D.M., Arras K.O. (2020). Human motion trajectory prediction: A survey. Int. J. Robot. Res..

[86] Schubert R., Richter E., Wanielik G. Comparison and evaluation of advanced motion models for vehicle tracking. Proceedings of the 11th International Conference on Information Fusion.

[87] Yamaguchi K., Berg A.C., Ortiz L.E., Berg T.L. Who are you with and where are you going?. Proceedings of the CVPR 2011.

[88] Pellegrini S., Ess A., Schindler K., van Gool L. You’ll never walk alone: Modeling social behavior for multi-target tracking. Proceedings of the IEEE 12th International Conference on Computer Vision.

[89] Kooij J.F., Flohr F., Pool E.A., Gavrila D.M. (2019). Context-based path prediction for targets with switching dynamics. Int. J. Comput. Vis..

[90] Shi X., Shao X., Fan Z., Jiang R., Zhang H., Guo Z., Wu G., Yuan W., Shibasaki R. Multimodal Interaction-Aware Trajectory Prediction in Crowded Space. Proceedings of the AAAI Conference on Artificial Intelligence.

[91] Huang Y., Bi H., Li Z., Mao T., Wang Z. Stgat: Modeling spatial-temporal interactions for human trajectory prediction. Proceedings of the IEEE/CVF International Conference on Computer Vision.

[92] Alahi A., Goel K., Ramanathan V., Robicquet A., Li F.F., Savarese S. Social lstm: Human trajectory prediction in crowded spaces. Proceedings of the IEEE Conference on Computer Vision and Pattern Recognition.

[93] Xue H., Huynh D.Q., Reynolds M. SS-LSTM: A Hierarchical LSTM Model for Pedestrian Trajectory Prediction. Proceedings of the IEEE Winter Conference on Applications of Computer Vision (WACV).

[94] Goldhammer M., Doll K., Brunsmann U., Gensler A., Sick B. Pedestrian’s trajectory forecast in public traffic with artificial neural networks. Proceedings of the 22nd International Conference on Pattern Recognition.

[95] Xiao S., Wang Z., Folkesson J. Unsupervised robot learning to predict person motion. Proceedings of the IEEE International Conference on Robotics and Automation (ICRA).

[96] Luber M., Spinello L., Silva J., Arras K.O. Socially-aware robot navigation: A learning approach. Proceedings of the IEEE/RSJ International Conference on Intelligent Robots and Systems.

[97] Rösmann C., Oeljeklaus M., Hoffmann F., Bertram T. Online trajectory prediction and planning for social robot navigation. Proceedings of the IEEE International Conference on Advanced Intelligent Mechatronics (AIM).

[98] Vasquez D. Novel planning-based algorithms for human motion prediction. Proceedings of the IEEE International Conference on Robotics and Automation (ICRA).

[99] Karasev V., Ayvaci A., Heisele B., Soatto S. Intent-aware long-term prediction of pedestrian motion. Proceedings of the IEEE International Conference on Robotics and Automation (ICRA).

[100] Shen M., Habibi G., How J.P. Transferable pedestrian motion prediction models at intersections. Proceedings of the IEEE/RSJ International Conference on Intelligent Robots and Systems (IROS).

[101] Pfeiffer M., Schwesinger U., Sommer H., Galceran E., Siegwart R. Predicting actions to act predictably: Cooperative partial motion planning with maximum entropy models. Proceedings of the 2016 IEEE/RSJ International Conference on Intelligent Robots and Systems (IROS).

[102] Chung S.Y., Huang H.P. Incremental learning of human social behaviors with feature-based spatial effects. Proceedings of the IEEE/RSJ International Conference on Intelligent Robots and Systems.

[103] Bera A., Randhavane T., Manocha D. The emotionally intelligent robot: Improving socially-aware human prediction in crowded environments. Proceedings of the IEEE/CVF Conference on Computer Vision and Pattern Recognition Workshops.

[104] Wang Z., Jensfelt P., Folkesson J. Building a human behavior map from local observations. Proceedings of the 25th IEEE International Symposium on Robot and Human Interactive Communication (RO-MAN).

[105] Robicquet A., Sadeghian A., Alahi A., Savarese S. (2016). Learning social etiquette: Human trajectory understanding in crowded scenes. Proceedings of the European Conference on Computer Vision.

[106] Rios-Martinez J., Spalanzani A., Laugier C. (2015). From proxemics theory to socially-aware navigation: A survey. Int. J. Soc. Robot..

[107] Charalampous K., Kostavelis I., Gasteratos A. (2017). Recent trends in social aware robot navigation: A survey. Robot. Auton. Syst..

[108] Kruse T., Pandey A.K., Alami R., Kirsch A. (2013). Human-aware robot navigation: A survey. Robot. Auton. Syst..

[109] Möller R., Furnari A., Battiato S., Härmä A., Farinella G.M. (2021). A Survey on Human-aware Robot Navigation. arXiv.

[110] Cheng J., Cheng H., Meng M.Q.H., Zhang H. Autonomous navigation by mobile robots in human environments: A survey. Proceedings of the IEEE International Conference on Robotics and Biomimetics (ROBIO).

[111] Hall E.T., Birdwhistell R.L., Bock B., Bohannan P., Diebold A.R., Durbin M., Edmonson M.S., Fischer J., Hymes D., Kimball S.T. (1968). Proxemics [and comments and replies]. Curr. Anthropol..

[112] Tai L., Zhang J., Liu M., Burgard W. Socially Compliant Navigation Through Raw Depth Inputs with Generative Adversarial Imitation Learning. Proceedings of the IEEE International Conference on Robotics and Automation (ICRA).

[113] Choi S., Kim E., Lee K., Oh S. (2017). Real-time nonparametric reactive navigation of mobile robots in dynamic environments. Robot. Auton. Syst..

[114] Guy S.J., Lin M.C., Manocha D. (2010). Modeling collision avoidance behavior for virtual humans. Auton. Agents Multiagent Syst..

[115] Li K., Xu Y., Wang J., Meng M.Q.H. SARL*: Deep Reinforcement Learning based Human-Aware Navigation for Mobile Robot in Indoor Environments. Proceedings of the IEEE International Conference on Robotics and Biomimetics (ROBIO).

[116] SLAMTEC RPLiDAR-A2. https://www.slamtec.com/en/Lidar/A2.

[117] Aoude G.S., Luders B.D., Joseph J.M., Roy N., How J.P. (2013). Probabilistically safe motion planning to avoid dynamic obstacles with uncertain motion patterns. Auton. Robot..

[118] Lu D.V., Smart W.D. Towards more efficient navigation for robots and humans. Proceedings of the IEEE/RSJ International Conference on Intelligent Robots and Systems.

[119] Vemula A., Muelling K., Oh J. Modeling cooperative navigation in dense human crowds. Proceedings of the IEEE International Conference on Robotics and Automation (ICRA), Marina Bay Sands.

[120] Chen C., Liu Y., Kreiss S., Alahi A. Crowd-Robot Interaction: Crowd-Aware Robot Navigation With Attention-Based Deep Reinforcement Learning. Proceedings of the International Conference on Robotics and Automation (ICRA).

[121] Bera A., Randhavane T., Prinja R., Manocha D. SocioSense: Robot navigation amongst pedestrians with social and psychological constraints. Proceedings of the IEEE/RSJ International Conference on Intelligent Robots and Systems (IROS).

[122] Truong X.T., Ngo T.D. (2017). Toward Socially Aware Robot Navigation in Dynamic and Crowded Environments: A Proactive Social Motion Model. IEEE Trans. Autom. Sci. Eng..

[123] Liu Y., Fu Y., Chen F., Goossens B., Tao W., Zhao H. (2021). Simultaneous Localization and Mapping Related Datasets: A Comprehensive Survey. arXiv.

[124] Ramezani M., Wang Y., Camurri M., Wisth D., Mattamala M., Fallon M. The Newer College Dataset: Handheld LiDAR, Inertial and Vision with Ground Truth. Proceedings of the IEEE/RSJ International Conference on Intelligent Robots and Systems (IROS).

[125] Funk N., Tarrio J., Papatheodorou S., Popović M., Alcantarilla P.F., Leutenegger S. (2021). Multi-resolution 3D mapping with explicit free space representation for fast and accurate mobile robot motion planning. IEEE Robot. Autom. Lett..

[126] Liang S., Cao Z., Wang C., Yu J. (2020). A Novel 3D LiDAR SLAM Based on Directed Geometry Point and Sparse Frame. IEEE Robot. Autom. Lett..

[127] Cowley A., Miller I.D., Taylor C.J. (2021). UPSLAM: Union of panoramas SLAM. arXiv.

[128] Wang Y., Funk N., Ramezani M., Papatheodorou S., Popovic M., Camurri M., Leutenegger S., Fallon M. (2020). Elastic and efficient LiDAR reconstruction for large-scale exploration tasks. arXiv.

[129] Wang Y., Ramezani M., Mattamala M., Fallon M. (2021). Scalable and Elastic LiDAR Reconstruction in Complex Environments Through Spatial Analysis. arXiv.

[130] Amblard V., Osedach T.P., Croux A., Speck A., Leonard J.J. (2021). Lidar-Monocular Surface Reconstruction Using Line Segments. arXiv.

[131] Geiger A., Lenz P., Stiller C., Urtasun R. (2013). Vision meets Robotics: The KITTI Dataset. Int. J. Robot. Res. IJRR.

[132] Deschaud J.E. IMLS-SLAM: Scan-to-model matching based on 3D data. Proceedings of the IEEE International Conference on Robotics and Automation (ICRA).

[133] Chen X., Milioto A., Palazzolo E., Giguere P., Behley J., Stachniss C. Suma++: Efficient lidar-based semantic slam. Proceedings of the IEEE/RSJ International Conference on Intelligent Robots and Systems (IROS).

[134] Chen X., Läbe T., Milioto A., Röhling T., Vysotska O., Haag A., Behley J., Stachniss C. (2021). OverlapNet: Loop closing for LiDAR-based SLAM. arXiv.

[135] Engel J., Stückler J., Cremers D. Large-scale direct SLAM with stereo cameras. Proceedings of the IEEE/RSJ International Conference on Intelligent Robots and Systems (IROS).

[136] Shin Y.S., Park Y.S., Kim A. Direct visual slam using sparse depth for camera-lidar system. Proceedings of the IEEE International Conference on Robotics and Automation (ICRA).

[137] Gomez-Ojeda R., Moreno F.A., Zuniga-Noël D., Scaramuzza D., Gonzalez-Jimenez J. (2019). PL-SLAM: A stereo SLAM system through the combination of points and line segments. IEEE Trans. Robot..

[138] Brasch N., Bozic A., Lallemand J., Tombari F. Semantic monocular SLAM for highly dynamic environments. Proceedings of the IEEE/RSJ International Conference on Intelligent Robots and Systems (IROS).

[139] Ling Y., Shen S. Building maps for autonomous navigation using sparse visual SLAM features. Proceedings of the IEEE/RSJ International Conference on Intelligent Robots and Systems (IROS).

[140] Jaramillo-Avila U., Aitken J.M., Anderson S.R. Visual saliency with foveated images for fast object detection and recognition in mobile robots using low-power embedded GPUs. Proceedings of the 19th International Conference on Advanced Robotics (ICAR).

[141] Zhang J., Liang X., Wang M., Yang L., Zhuo L. (2020). Coarse-to-fine object detection in unmanned aerial vehicle imagery using lightweight convolutional neural network and deep motion saliency. Neurocomputing.

[142] Girisha S., Pai M.M., Verma U., Pai R.M. (2019). Performance analysis of semantic segmentation algorithms for finely annotated new uav aerial video dataset (manipaluavid). IEEE Access.

[143] Cengiz E., Yılmaz C., Kahraman H.T., Bayram F. Pedestrian and Vehicles Detection with ResNet in Aerial Images. Proceedings of the 4th International Symposium on Innovative Approaches in Engineering and Natural Sciences.

[144] Cengiz E., Yilmaz C., Kahraman H. (2021). Classification of Human and Vehicles with the Deep Learning Based on Transfer Learning Method. Düzce Üniv. Bilim. Teknol. Derg..

[145] Sadeghian A., Kosaraju V., Sadeghian A., Hirose N., Rezatofighi H., Savarese S. Sophie: An attentive gan for predicting paths compliant to social and physical constraints. Proceedings of the IEEE/CVF Conference on Computer Vision and Pattern Recognition.

[146] Mohamed A., Qian K., Elhoseiny M., Claudel C. Social-stgcnn: A social spatio-temporal graph convolutional neural network for human trajectory prediction. Proceedings of the IEEE/CVF Conference on Computer Vision and Pattern Recognition.

[147] Bock J., Krajewski R., Moers T., Runde S., Vater L., Eckstein L. The ind dataset: A drone dataset of naturalistic road user trajectories at german intersections. Proceedings of the IEEE Intelligent Vehicles Symposium (IV).

[148] Ridel D., Deo N., Wolf D., Trivedi M. (2020). Scene compliant trajectory forecast with agent-centric spatio-temporal grids. IEEE Robot. Autom. Lett..

[149] Chai Y., Sapp B., Bansal M., Anguelov D. (2019). Multipath: Multiple probabilistic anchor trajectory hypotheses for behavior prediction. arXiv.

[150] Poibrenski A., Klusch M., Vozniak I., Müller C. (2021). Multimodal multi-pedestrian path prediction for autonomous cars. ACM SIGAPP Appl. Comput. Rev..

[151] Lai W.C., Xia Z.X., Lin H.S., Hsu L.F., Shuai H.H., Jhuo I.H., Cheng W.H. Trajectory prediction in heterogeneous environment via attended ecology embedding. Proceedings of the 28th ACM International Conference on Multimedia.

[152] Chen X.Z., Liu C.Y., Yu C.W., Lee K.F., Chen Y.L. A Trajectory Prediction Method Based on Social Forces, Scene Information and Motion Habit. Proceedings of the IEEE International Conference on Consumer Electronics (ICCE).

[153] Hu Y., Chen S., Zhang Y., Gu X. Collaborative motion prediction via neural motion message passing. Proceedings of the IEEE/CVF Conference on Computer Vision and Pattern Recognition.

[154] Fragkiadaki K., Huang J., Alemi A., Vijayanarasimhan S., Ricco S., Sukthankar R. (2017). Motion prediction under multimodality with conditional stochastic networks. arXiv.

[155] Monti A., Bertugli A., Calderara S., Cucchiara R. Dag-net: Double attentive graph neural network for trajectory forecasting. Proceedings of the 25th International Conference on Pattern Recognition (ICPR).

[156] Li J., Ma H., Tomizuka M. Conditional generative neural system for probabilistic trajectory prediction. Proceedings of the IEEE/RSJ International Conference on Intelligent Robots and Systems (IROS).

[157] Ess A., Leibe B., Schindler K., van Gool L. A Mobile Vision System for Robust Multi-Person Tracking. Proceedings of the IEEE Conference on Computer Vision and Pattern Recognition (CVPR’08).

[158] Gupta A., Johnson J., Fei-Fei L., Savarese S., Alahi A. Social gan: Socially acceptable trajectories with generative adversarial networks. Proceedings of the IEEE Conference on Computer Vision and Pattern Recognition.

[159] Liang J., Jiang L., Niebles J.C., Hauptmann A.G., Fei-Fei L. Peeking into the future: Predicting future person activities and locations in videos. Proceedings of the IEEE/CVF Conference on Computer Vision and Pattern Recognition.

[160] Mangalam K., Girase H., Agarwal S., Lee K.H., Adeli E., Malik J., Gaidon A., Vedaldi A., Bischof H., Brox T., Frahm J.M. (2020). It Is Not the Journey However, the Destination: Endpoint Conditioned Trajectory Prediction. Computer Vision—ECCV 2020.

[161] Jia D., Steinweg M., Hermans A., Leibe B. Self-Supervised Person Detection in 2D Range Data using a Calibrated Camera. Proceedings of the 2021 IEEE International Conference on Robotics and Automation (ICRA).

[162] Linder T., Vaskevicius N., Schirmer R., Arras K.O. Cross-Modal Analysis of Human Detection for Robotics: An Industrial Case Study. Proceedings of the 2021 IEEE/RSJ International Conference on Intelligent Robots and Systems (IROS).

[163] Caselles-Dupré H., Garcia-Ortiz M., Filliat D. (2020). On the sensory commutativity of action sequences for embodied agents. arXiv.

[164] Jia D., Leibe B. (2021). Person-MinkUNet: 3D Person Detection with LiDAR Point Cloud. arXiv.

[165] Jia D., Hermans A., Leibe B. (2021). Domain and Modality Gaps for LiDAR-based Person Detection on Mobile Robots. arXiv.

[166] Tolani V., Bansal S., Faust A., Tomlin C. (2021). Visual navigation among humans with optimal control as a supervisor. IEEE Robot. Autom. Lett..

[167] Caesar H., Bankiti V., Lang A.H., Vora S., Liong V.E., Xu Q., Krishnan A., Pan Y., Baldan G., Beijbom O. (2019). nuScenes: A multimodal dataset for autonomous driving. arXiv.

[168] Yin T., Zhou X., Krahenbuhl P. Center-based 3d object detection and tracking. Proceedings of the IEEE/CVF Conference on Computer Vision and Pattern Recognition.

[169] Zhu X., Ma Y., Wang T., Xu Y., Shi J., Lin D. (2020). Ssn: Shape signature networks for multi-class object detection from point clouds. Proceedings of the Computer Vision—ECCV 2020: 16th European Conference.

[170] Guo Y., Wang H., Hu Q., Liu H., Liu L., Bennamoun M. (2021). Deep Learning for 3D Point Clouds: A Survey. IEEE Trans. Pattern Anal. Mach. Intell..

[171] Nabati R., Qi H. Centerfusion: Center-based radar and camera fusion for 3d object detection. Proceedings of the IEEE/CVF Winter Conference on Applications of Computer Vision.

[172] Zhou X., Koltun V., Krähenbühl P. (2020). Tracking objects as points. Proceedings of the European Conference on Computer Vision.

[173] Rasouli A., Yau T., Rohani M., Luo J. (2020). Multi-modal hybrid architecture for pedestrian action prediction. arXiv.

[174] Yau T., Malekmohammadi S., Rasouli A., Lakner P., Rohani M., Luo J. Graph-sim: A graph-based spatiotemporal interaction modelling for pedestrian action prediction. Proceedings of the 2021 IEEE International Conference on Robotics and Automation (ICRA).

[175] Yuan Y., Weng X., Ou Y., Kitani K. (2021). AgentFormer: Agent-Aware Transformers for Socio-Temporal Multi-Agent Forecasting. arXiv.

